# A bacteria colony-based screen for optimal linker combinations in genetically encoded biosensors

**DOI:** 10.1186/1472-6750-11-105

**Published:** 2011-11-10

**Authors:** Andreas Ibraheem, Hongkin Yap, Yidan Ding, Robert E Campbell

**Affiliations:** 1 Department of Chemistry, University of Alberta, Edmonton, Alberta T6G 2G2, Canada; 2 The Hong Kong Polytechnic University, Hung Hom, Kowloon, Hong Kong, People's Republic of China

## Abstract

**Background:**

Fluorescent protein (FP)-based biosensors based on the principle of intramolecular Förster resonance energy transfer (FRET) enable the visualization of a variety of biochemical events in living cells. The construction of these biosensors requires the genetic insertion of a judiciously chosen molecular recognition element between two distinct hues of FP. When the molecular recognition element interacts with the analyte of interest and undergoes a conformational change, the ratiometric emission of the construct is altered due to a change in the FRET efficiency. The sensitivity of such biosensors is proportional to the change in ratiometric emission, and so there is a pressing need for methods to maximize the ratiometric change of existing biosensor constructs in order to increase the breadth of their utility.

**Results:**

To accelerate the development and optimization of improved FRET-based biosensors, we have developed a method for function-based high-throughput screening of biosensor variants in colonies of *Escherichia coli*. We have demonstrated this technology by undertaking the optimization of a biosensor for detection of methylation of lysine 27 of histone H3 (H3K27). This effort involved the construction and screening of 3 distinct libraries: a domain library that included several engineered binding domains isolated by phage-display; a lower-resolution linker library; and a higher-resolution linker library.

**Conclusion:**

Application of this library screening methodology led to the identification of an optimized H3K27-trimethylation biosensor that exhibited an emission ratio change (66%) that was 2.3 × improved relative to that of the initially constructed biosensor (29%).

## Background

By providing researchers with a means of genetically encoding fluorescence, fluorescent proteins (FPs) have essentially turned mammalian cells into living test tubes for performing many types of biochemical assays. One of the most sophisticated applications of FPs is their use in the construction of proteinaceous biosensors for a variety of enzyme activities in live cells [1]. A biosensor design strategy that has proven to be particularly robust and versatile is the modulation of Förster resonance energy transfer (FRET) efficiency between a blue shifted donor FP and a red shifted acceptor FP [2]. The key to creation of such biosensors is that a protein containing both a donor and an acceptor FP undergoes an enzyme activity-dependent conformational change such that the distance and/or fluorophore dipole orientation between the FPs is modified [3]. This change in distance or orientation results in a change in FRET efficiency that manifests itself as a change in emission ratio.

Although the designs principles of FRET-based biosensors are relatively well-established [1], methods for optimizing the signal-to-noise ratio of the FRET change are not. The goal of any optimization effort is to maximize the ratio change between the initial and final states of the biosensor by maximizing the change in distance and/or fluorophore dipole orientation [3]. Although some progress has been made in the computational prediction of FRET changes [4], empirical screening remains the most effective method of achieving substantial improvements. Previous optimization efforts have involved the tedious and systematic modification of the linkers, topology, and domain identities [5-7]. In one of the single most exhaustive efforts to optimize a FRET based biosensor, 176 systematically varied linker combinations of a glutamate biosensor were constructed and individually tested *in vitro *to identify the one with the highest ratio change [7]. The position in 'linker space' and the magnitude of ratio change did not follow any predictable trend and only one of the 176 linker combinations exhibited a substantial increase in ratio change. Clearly, rapid and high-throughput means for optimizing combinations of two or three linkers in FRET-based biosensors could accelerate the development of improved tools for both basic biochemical and applied pharmaceutical research.

Inspired by the fact that fluorescence screening in bacterial colonies has been the technology of choice for the directed evolution of improved FPs, we sought to extend this methodology to the screening of biosensors. However, unlike individual FPs that have a static and unchanging fluorescence, biosensors have a dynamic fluorescence emission that must be imaged in both its initial baseline state and its final stimulated state. Accordingly, the primary challenge of screening biosensors in bacterial colonies is how to induce the biochemical change (*e.g*., onset of an enzyme activity or a change in small molecule concentration), that the biosensor is designed to sense. To address this challenge we have developed a screening system in which the functional response of a FRET-based biosensor for a post-translational modification can be artificially induced in live bacterial colonies. We note that an alternative approach to addressing this challenge is to optimize a FRET-based biosensor in mammalian cells. In recent work, Piljić et al. have used this alternative approach to optimize the FRET response of biosensors for detection of the activation of two calcium/calmodulin-dependent kinases [8]. The advantage of this approach is that the sensor is optimized for use in the same context in which it will ultimately be applied. A disadvantage is that the throughput of the screening approach is substantially less than what can be achieved using bacterial colonies.

**Figure 1:**
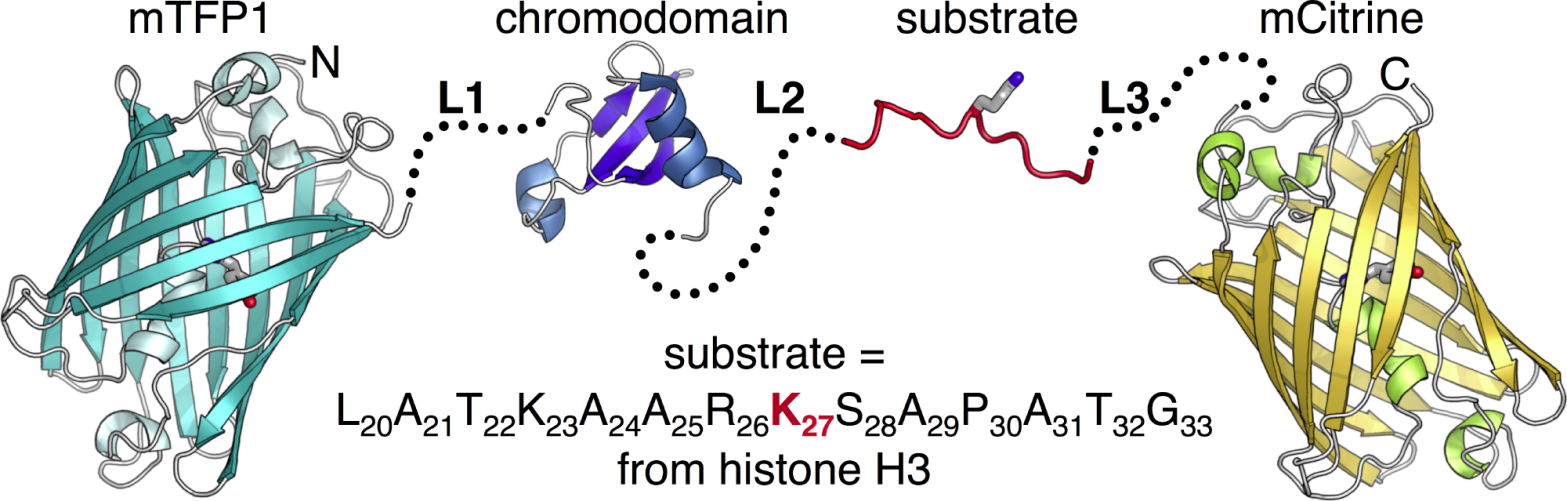
**Representation of the H3K27-trimethylation biosensor (**H3K27-MetBio**)**. The biosensor is a four-part genetic chimera composed of the FRET donor mTFP1 [18], a domain that binds to methylated lysine residues, residues 20 to 33 of histone H3 (GenBank: CAA40407), and the FRET acceptor mCitrine [37]. The first (L1), second (L2), and third (L3) linkers are represented with dotted lines. L1 starts immediately after residue 225 of mTFP1 and ends immediately before residue 1 of the chromodomain. L2 starts immediately after the last residue of the chromodomain and ends immediately before residue 21 of the histone H3-derived substrate. L3 starts immediately after the last residue of the substrate and ends immediately before residue 6 of mCitrine. This biosensor design has been previously reported [9].

As a model system for demonstrating the utility of screening in bacterial colonies, we have undertaken the optimization of a biosensor for enzymatic trimethylation of lysine 27 of histone H3 (H3K27) (Figure 1). A previously reported biosensor of this enzyme activity [9], which was rationally designed based on x-ray crystallographic information [10,11] and not empirically optimized, had a ratio change of 28%. As a similar biosensor for methylation of lysine 9 of histone H3 (H3K9) had a ratio change of 60% [9], it seemed reasonable that further improvements of a H3K27-trimethylation biosensor (hereafter abbreviated as H3K27-MetBio) could be achieved with linker optimization. Another important consideration was the availability of the *Paramecium bursaria *chlorella virus SET domain protein (vSET) that catalyzes H3K27-trimethylation and can be produced in soluble and active form in *E. coli *[12,13].

## Results and discussion

### Design and optimization of a colony-based screening system

**Figure 2:**
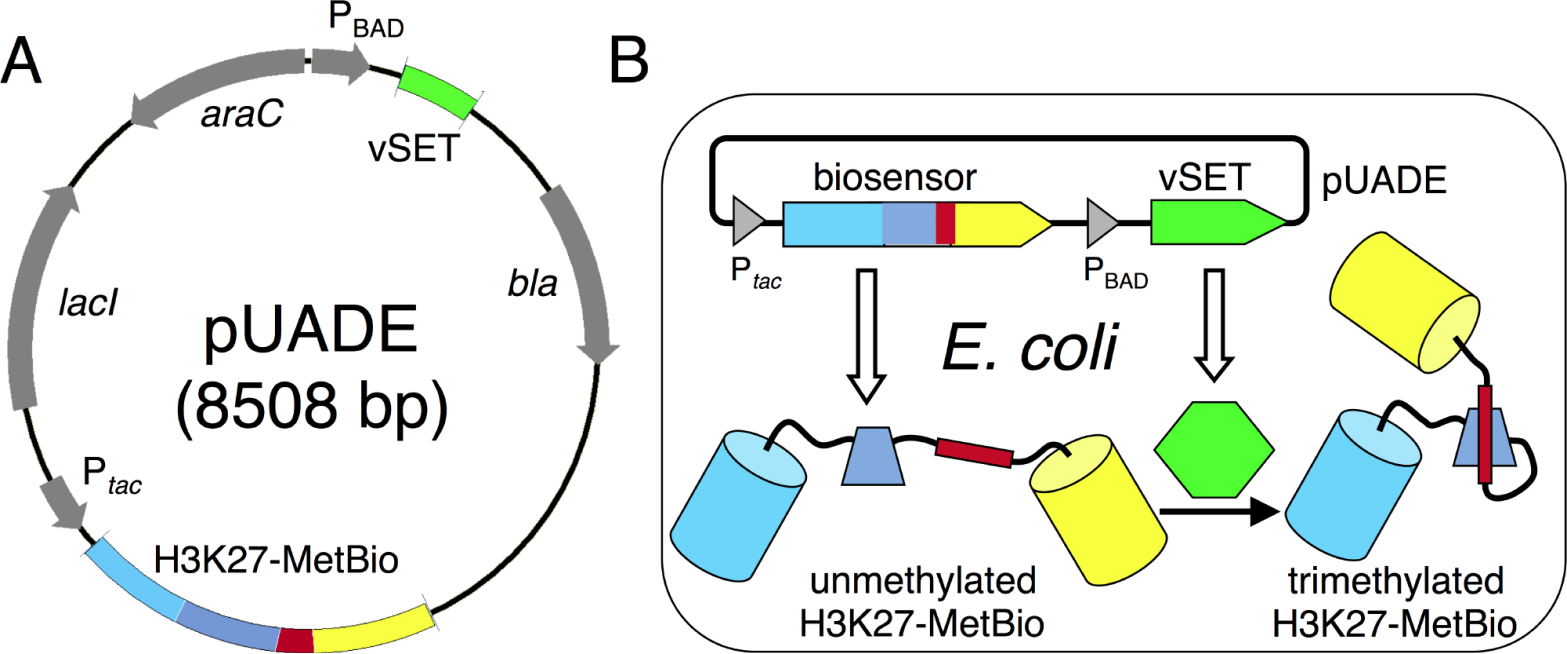
**A dual expression system for induced post-translational modification of a genetically encoded biosensor**. (A) Plasmid map of pUADE. P*_tac _*is an IPTG-inducible promoter and P_BAD _is an L-arabinose-inducible promoter. (B) Schematic representation of protein expression from pUADE in *E. coli *in the presence of both IPTG and L-arabinose. In the absence of L-arabinose, the P_BAD _promoter is inactive and no vSET is produced.

To achieve the inducible post-translational modification of a H3K27-MetBio in *E. coli*, we designed a dual expression plasmid (designated pUADE) that expresses the biosensor construct under control of the *tac *promoter (P*_tac_*) [14] and vSET under control of the *ara*BAD promoter (P_BAD_) [15] (Figure 2A). We reasoned that a single-plasmid based approach would simplify the experimental procedures by eliminating the need for multiple transformations and more than one resistance marker. The P_BAD _promoter was chosen because it has been reported to provide tight regulation of gene expression [16] and would allow us to use L-arabinose to selectively "turn on" vSET expression in colonies of *E. coli *that are also expressing the biosensor (Figure 2B).

**Figure 3:**
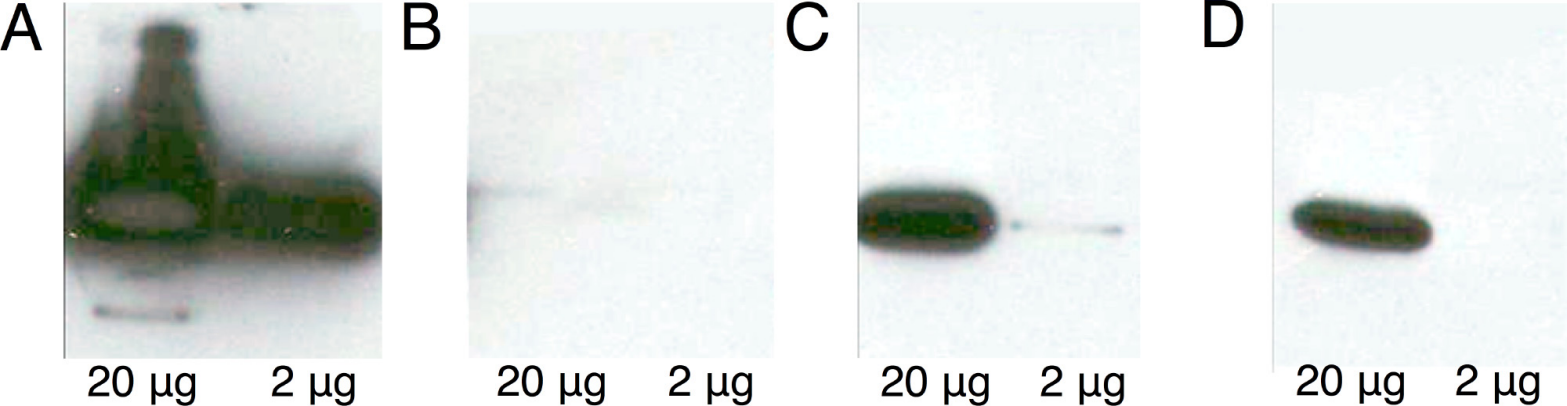
**Optimization of expression conditions**. Western blots showing expression of vSET for colonies of *E. coli *grown in the presence or absence of inducer. For each condition, 20 μg of total protein was loaded in the left lane and 2 μg was loaded in the right lane. (A) Media containing IPTG (1 mM) and L-arabinose (13 mM). (B) Media containing IPTG (1 mM) and D-glucose (11 mM). (C) Media conditions identical to (B), 3 h after being sprayed with a concentrated solution of L-arabinose. (D) Media containing IPTG (1 mM).

To determine the level of induction that we could achieve for vSET under the P_BAD _promoter, in the context of *E. coli *colonies grown under conditions that would also induce P*_tac_*, we used both Western blot analysis and fluorescence imaging of bacterial colonies. As shown in Figure 3A-D, Western blot analysis with an anti-His-tag antibody was used to qualitatively determine the relative abundance of vSET (in pBAD/His B) in *E. coli *grown on media containing various concentrations of D-glucose, IPTG, and L-arabinose. As expected, vSET expression was very strong under the inducing conditions (Figure 3A; with L-arabinose), but was not detectable under the repressive conditions (Figure 3B; with D-glucose). Spraying of the colonies grown under the repressive conditions with a concentrated solution of L-arabinose (1 M) resulted in a substantial increase in the amount of vSET over a time period of 3 hours (Figure 3C), though the protein did not reach the same abundance as in the colonies grown in the presence of L-arabinose (compare to Figure 3A). If D-glucose was not included in the growth media, the P_BAD _promoter was not fully repressed as indicated by the detection of vSET protein (Figure 3D). We noted that colonies grown on media that contains L-arabinose and IPTG, but no D-glucose, tended to be relatively small, suggesting an increased metabolic burden on the *E. coli *[17].

**Figure 4:**
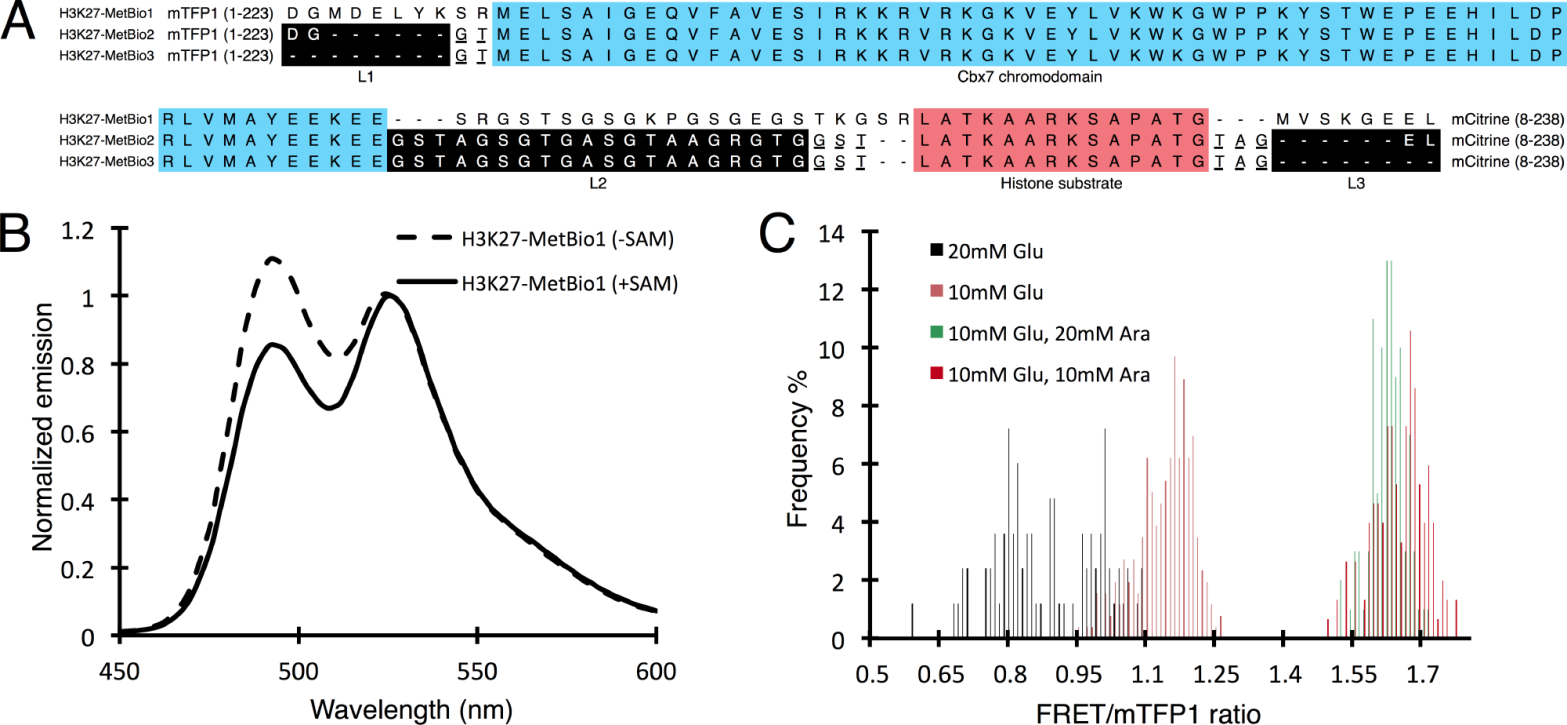
**Structure and characterization of H3K27-MetBio1**. (A) Sequence alignment of H3K27-MetBio1, H3K27-MetBio2, and H3K27-MetBio3. Underlined residues in the H3K27-MetBio2 and H3K27-MetBio3 sequences denote amino acids encoded by restriction sites for Kpn1 (GT), Sal1 (GST), and Eag1 (TAG). (B) Emission spectra of the H3K27-MetBio1 protein in both the methylated and unmethylated state, normalized to acceptor intensity. (C) Image-based emission ratios for H3K27-MetBio1 in colonies of *E. coli *that were grown on different plate compositions. All plates contained 1 mM IPTG.

Having confirmed that vSET expression could be "turned on" in colonies by treating with L-arabinose, we next tested whether we could induce a change in the FRET emission signal of a H3K27-MetBio expressed in colonies of *E. coli*. Accordingly, we constructed the pUADE plasmid with vSET under the P_BAD _promoter and a first generation H3K27-MetBio (H3K27-MetBio1) under the P*_tac _*promoter. In contrast to the previously reported H3K27 biosensor [9] which incorporated the CFP-YFP FRET pair and the Polycomb chromodomain [10,11], H3K27-MetBio1 incorporated the mTFP1-mCitrine FRET pair [18] and the Cbx7 chromodomain [19] (Figure 1). Cbx7 has high homology with Polycomb and exhibits a strong preference for binding to trimethylated H3K27 over the di-, mono-, and unmethylated forms [19]. The choice of the Cbx7 chromodomain was later revealed to be fortuitous since this binding domain produced the largest FRET ratio change of all domains tested. CFP was substituted with mTFP1 due to the improved brightness and photostability of the latter protein [18]. Following our convention for linker length description (refer to Figure 1 legend), L1, L2, and L3 of H3K27-MetBio1 consisted of 8, 22, and 6 amino acids respectively. The sequences of L1 and L3 corresponded to the normal C- and N-terminal sequences of the respective FPs in addition to the amino acids encoded by the codons of the restriction sites (in case of L1). L2 corresponded to a 18-residue unstructured linker [20] flanked by the two amino acids at each end that are encoded by the restriction sites. The complete sequence of H3K27-MetBio1 and other key variants is provided in Figure 4A.

*In vitro *characterization of H3K27-MetBio1 revealed that this construct exhibited a 29% change in emission ratio change upon treatment with vSET in the presence of *S*-adenosyl methionine (SAM) (Figure 4B). To determine if methylation of H3K27-MetBio1 was also occurring in *E. coli *under conditions where vSET was being expressed, we transformed cells with the pUADE-(vSET; H3K27-MetBio1) plasmid and cultured colonies (100s per plate) on media supplemented with 1 mM IPTG plus various combinations of L-arabinose and D-glucose. After allowing colonies to grow overnight at 37°C, plates were imaged using a fluorescence imaging system equipped with bandpass filters that allowed us to acquire fluorescence images of the mTFP1 donor (426-446 nm excitation and 460-500 nm emission) and the acceptor fluorescence due to FRET (426-446 nm excitation and 520-550 nm emission) [21]. Processing of the digital images provided the average acceptor to donor (*i.e*., FRET/mTFP1) ratios for the colonies. As shown in Figure 4C, the FRET/mTFP1 ratios for colonies grown in the presence of L-arabinose and D-glucose were substantially higher than those grown in the presence of D-glucose alone. This result provided strong support for the conclusion that H3K27-MetBio1 could be methylated by recombinant vSET in the context of *E. coli *colonies. For all future experiments we used 20 mM D-glucose as our repressing condition (hereafter, 'glucose' plates) and 10 mM L-arabinose as our inducing condition ('arabinose' plates). Both the glucose and arabinose plates contained 1 mM IPTG to induce the production of H3K27-MetBio. Unlike the very slow growing colonies on plates containing only L-arabinose and IPTG, colonies grown on media with both L-arabinose and D-glucose grew at a rate that was comparable to colonies grown on media containing only D-glucose. Presumably, adding D-glucose decreased vSET expression to a level that is less metabolically burdensome.

### Library screening identifies biosensors with increased FRET changes

**Figure 5:**
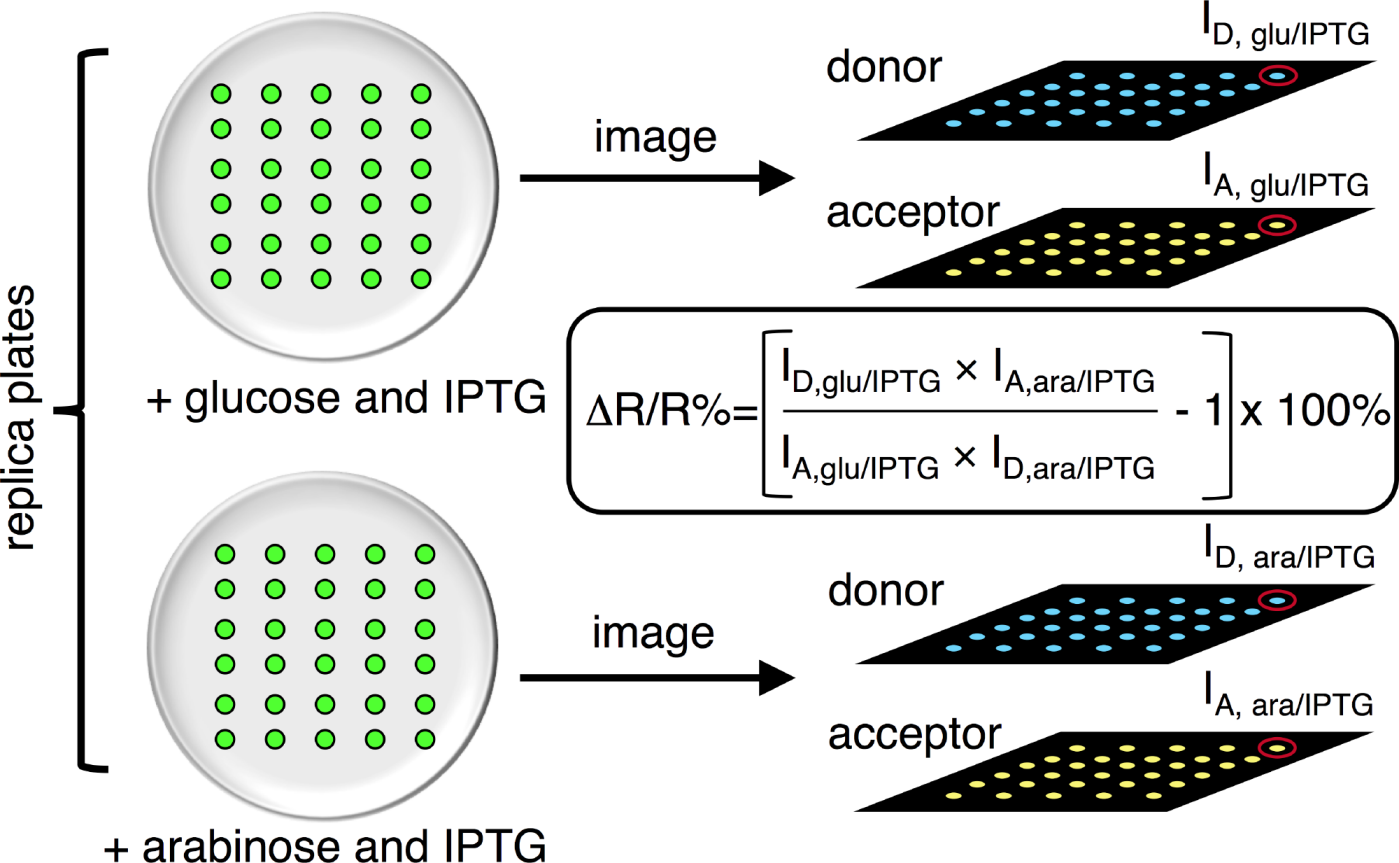
**Library screening methodology**. To screen libraries of biosensor variants for clones exhibiting improved ratio changes, an image-based method was used to determine FRET ratio changes in individual colonies of *E. coli *previously transformed with the plasmid library.

Having established that it was possible to methylate H3K27-MetBio1 in the context of bacterial colonies and then image the resulting change emission ratio for hundreds of individual colonies on a single plate, we next explored various methods of using this technology for library screening. Our goal was to identify the most robust and reliable procedure by which the emission ratio of a single clone could be determined under both inducing and repressing conditions for vSET expression. Seemingly, the ideal solution would be to image colonies on repressive media, spray with sufficient L-arabinose to induce vSET expression, and then image the same plate again. This approach proved challenging to implement due to the difficulty in getting a uniform application of spraying solution. Replica plating onto both inducing and repressing media seemed to offer an alternative solution, but ended up presenting new challenges that were ultimately insurmountable for us. Specifically, having missing colonies on one replicate was sufficient to make the digital processing steps effectively intractable, as the correlation between identical clones on the two different plates could not be automatically determined in software. We ultimately settled on the very robust, but more labor intensive, approach of manual plate replication by spotting of single colonies in two sets of ordered arrays. This approach achieved the goal of having identical clones cultured under both repressing and inducing conditions, and also greatly simplified the later digital image processing steps. A schematic representation of this library screening protocol is provided in Figure 5. Digital image processing using custom macros was used to extract the intensity for each colony on both replicate plates in both the donor and acceptor emission channels. Using the equation shown in Figure 5, the change in emission ratio for the transition from unmethylated to methylated biosensor (ΔR/R%) was calculated for each colony. Colonies exhibiting the highest ΔR/R% values were picked and cultured in order to provide plasmid DNA for sequencing.

Our overall strategy for optimizing the H3K27-MetBio involved the construction and subsequent screening of 3 iterative libraries; a domain library (lib1), a low resolution linker library (lib2), and a higher resolution linker library (lib3). Lib1 was kept quite small and consisted of just 7 distinct H3K27-MetBio1-derived variants, each of which had identical linkers but different binding domains. Four of the 7 binding domains were wild-type domains: amino acids 56-118 of CDY1 (chromodomain), accession AAD22735; amino acids 77-128 of C20orf140 (tudor domain), accession NP_057520; amino acids 890-1011 of JMJD2A (double tudor domain), accession NP_055478; and amino acids 36-97 of Cbx7 (chromodomain), accession EDL04620 [19,22]. The other 3 domains were variants of the wild-type domains that had been engineered for altered binding specificities towards trimethylated lysines: Cbx7 chromodomain A91K; JMJD2A double tudor domain D945K; and JMJD2A double tudor domain D945R. These engineered variants had been discovered by a phage display effort aimed at improving the binding specificity and/or affinity of these domains. As the gains in specificity and affinity were modest despite a substantial investment of effort, we did not further pursue the original goal. However, we did decide to include the best of the engineered variants in lib1 in the expectation that they could potentially alter the FRET response of the biosensor. Full details of the phage display effort is provided in Additional File 1.

For library screening, *E. coli *was transformed with the lib1 plasmid library and plated on regular LB/agar media. Individual colonies were picked at random and spotted onto both the inducing and repressing media at the same position within a regular grid. Approximately 24 h later, the colony grids were imaged as described above and colonies that exhibited the highest ΔR/R% values (a FRET increase) were picked, cultured, and their plasmid DNA isolated. DNA sequencing revealed that 9 contained Cbx7 and 1 contained Cbx7 A91K. The Cbx7 domain was not identified in clones that exhibited the lowest ΔR/R% values. This strong consensus clearly demonstrated that this screening approach could be used to identify H3K27-MetBio variants from colony-based libraries.

Having identified the preferred binding domain for H3K27-MetBio, our next goal was to find the combination of linkers that provided the optimal FRET ratio change. Accordingly, we constructed a second library (lib2) that was designed to contain 392 members that each had one of seven different lengths of L1 (ranging from 0 to 14 residues), eight different lengths of L2 (ranging from 0 to 20 residues), and seven different lengths for L3 (ranging from 0 to 14 residues). All linkers were composed primarily of glycine, alanine and serine, likely rendering them unstructured and highly flexible. By design, lib2 was relatively 'coarse' as our primary goal was to reveal the general trends in preferred linker length, such that we could eventually design a more refined second linker library.

**Figure 6:**
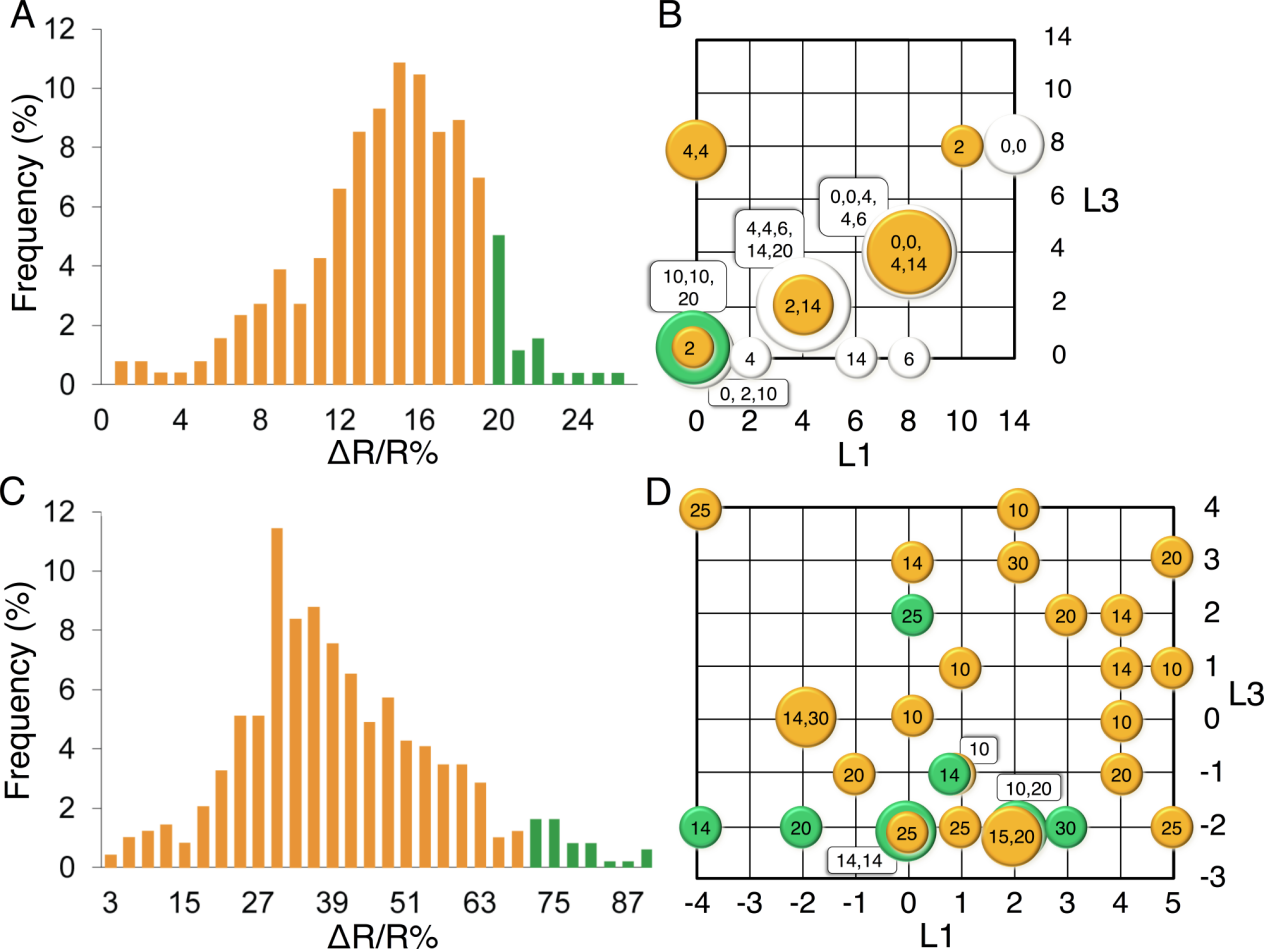
**Results of linker library screening**. (A) Histogram of FRET ratio changes of all colonies screened from lib2. The average FRET change in colonies was 14% with a standard deviation of 4%. (B) Linker combinations identified during screening of lib2. The horizontal and the vertical axes represent the number of amino acids constituting the first linker (L1) and the third linker (L3), respectively. The inputs for the second linker (L2) are sequences of 0, 2, 4, 6, 8, 10, 14, and 20 residues in length. Identified L1 and L3 combinations are indicated on the grid by coloured circles the size of which represents the number of sequenced clones with a given combination of L1 and L3, while the number of amino acids constituting L2 of these clones is indicated by the numerical values assigned to the circles. Linker combinations exhibiting the highest FRET ratio changes (> 20%) are represented by the green circle; orange circles represent clones with average-to-low ratio changes (< 20%); and grey circles represent clones picked at random. (C) Data histogram of FRET ratio changes of colonies screened from lib3. The average FRET change in colonies was 38% with a standard deviation of 16%. (D) Linker combinations identified during screening of lib3. Input for L2 are linkers of 6, 8, 10, 14, 15, 20, 25, or 30 residues in length. Linker combinations exhibiting high FRET ratio changes (> 70%) are represented with green circles and those showing average-to-low ratio changes (< 70%) are represented by orange circles.

**Figure 7:**
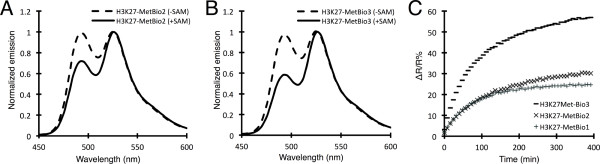
**Characterization of the optimized biosensor**. (A-B) Emission scans of H3K27-MetBio2 (A) and H3K27-MetBio3 (B) after treatment with vSET in the presence or absence of SAM, normalized to acceptor intensity. (C) Time course of FRET ratio changes for H3K27-MetBio variants treated with vSET and SAM and calculated using the ratio of fluorescence intensities at 526 nm and 494 nm.

To identify the best linker length combinations from lib2, six pairs of 'glucose' and 'arabinose' plates, each with 45 re-spotted colonies, were screened as shown in Figure 5. The average ratio change for all colonies was determined to be 14% with a standard deviation of 4% (Figure 6A). The three colonies which showed the highest FRET ratio changes; one which showed the lowest; and other 9 clones which showed average values were picked, cultured, and the H3K27-MetBio genes were sequenced. Sequencing results revealed that colonies which showed the highest ratio changes had L1 and L3 linker lengths of 0, and L2 linker lengths of 10, 14, or 20 (Figure 6B). The variant with L2 = 20 residues was designated as H3K27-MetBio2 (Figure 4A and 7A).

The results of lib2 screening suggested to us that the combination of a short L1, a long L2, and a short L3 linker would provide H3K27-MetBio variants with the highest FRET changes. This observed linker length pattern can be rationalized on the basis of the solution NMR structure of the Cbx7 chromodomain in complex with a trimethylated H3K27-containing peptide [23]. The structure reveals that the N-terminus of Cbx7 (to which mTFP1 is fused in H3K27-MetBio) is in close proximity to the C-terminus of the H3K27 peptide (to which mCitrine is fused in H3K27-MetBio). Accordingly, we expect that shorter L1 and L3 linkers, located at the N-terminus of Cbx7 and the C-terminus of the substrate peptide, respectively, could serve to hold the mTFP1-mCitrine FRET pair in closer proximity in the compacted (*i.e*., trimethylated) state of the biosensor and provide higher FRET efficiency than longer linkers would. In the extended (*i.e*., unmethylated) state of the biosensor, a longer L2 linker could serve to increase the overall distance between the FPs and provide a lower FRET efficiency than shorter linkers would. So overall, the observed linker combination is a reasonable solution to providing the maximal change in FRET efficiency upon methylation of H3K27-MetBio.

Guided by the results of the lib2 library screening, we attempted to further refine the H3K27-MetBio linkers by construction and screening of a second linker library designated lib3. The lib3 library consisted of 640 variants arising from the possible combination of ten L1 linkers (ranging from -4 to +5 residues), eight L2 linkers (ranging from 6 to 30 residues), and eight L3 linkers (ranging from -3 to +4 residues). Negative linkers indicate truncations relative to either Gly225 of mTFP1 in the case of L1 or relative to Glu6 of mCitrine in the case of L3.

For screening of lib3, twelve pairs of 'glucose' and 'arabinose' plates were prepared, and the FRET ratio changes of the colonies were calculated. The average ratio change was found to be 38% with a standard deviation of 16% (Figure 6C). Nine clones exhibiting high ratio changes (> 70%), and twenty-two with average-to-low ratio changes (< 70%) were picked for clonal expansion and further characterization. Sequencing results revealed that the L1 linker length in the high ratio change variants ranged from -4 to +3, indicating that there was no strong preference for any particular linker length within this range (Figure 6D). In contrast, 7 of 9 high ratio change variants had an L3 of -2, indicating that there is a much stronger dependence on this particular linker for achieving high ratio change. Similarly, 8 of the 9 clones with the highest ratio changes had L2 of 14 residues or longer. The variant with L1 = -2, L2 = 20, and L3 = -2 residues was designated as H3K27-MetBio3 (Figure 4A and 7B).

The best two variants identified from lib2 and lib3, H3K27-MetBio2 and H3K27-MetBio3, respectively, were purified in their unmodified states and a portion of the protein was subjected to an *in vitro *methylation assay. Fluorescence emission scans were acquired using an excitation wavelength of 420 nm (Figure 7AB) and ΔR/R% was calculated for each biosensor. H3K27-MetBio2 demonstrated only a modest improvement in signal change (37%) relative to the H3K27-MetBio1 (29%). However, H3K27-MetBio3 exhibited a substantially improved ΔR/R% of 66%, which is 2.3 × higher than H3K27-MetBio1. All 3 sensors were methylated by vSET at similar rates (Figure 7C).

### Imaging of chromatin-targeted H3K27-MetBio3

**Figure 8:**
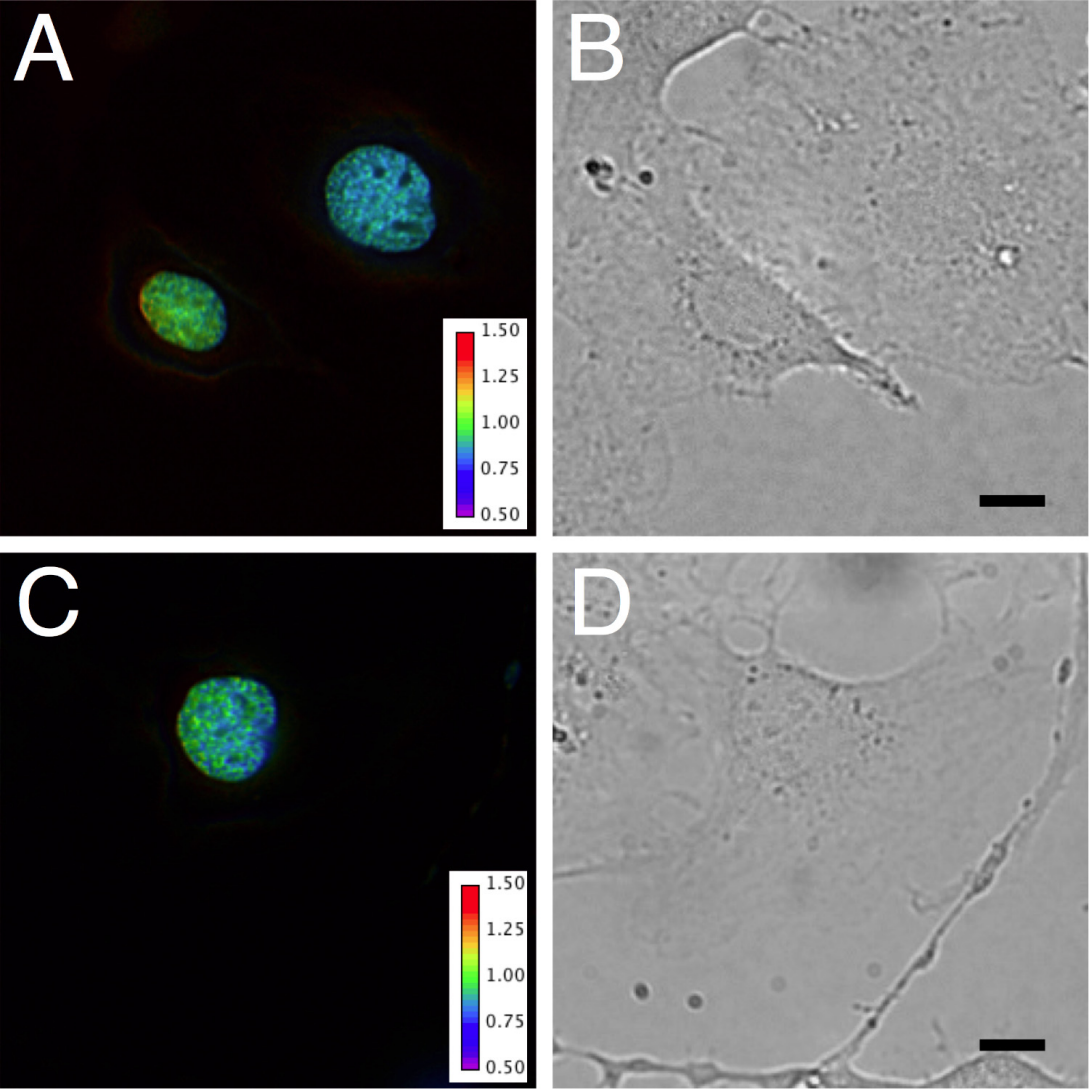
**Expression of H3K27-MetBio3 in mammalian cells**. (A and C) Representative ratiometric images of 3T3 cells expressing H3K27-MetBio3 as a fusion with histone 2B. (B and D) DIC transmitted light images of the same fields of view shown in A and C, respectively. Scale bar is 10 microns.

To investigate whether H3K27-MetBio3 could potentially be used to report on H3K27 trimethylation in the context of chromatin in living cells, we constructed a mammalian expression vector encoding a fusion of a histone 2B (H2B) and H3K27-MetBio3. Mouse embryonic fibroblast 3T3 cells transfected with this expression vector were viable and observed to proceed through mitosis. Ratiometric imaging of transfected cells revealed a speckle-like pattern of high FRET regions within the interphase chromatin (Figure 8) that is qualitatively similar to the pattern reported for immunofluorescence imaging with anti-H3K27 antibodies [24,25]. Further validation, potentially including immunostaining of transfected cells followed by imaging of colocalization with an antibody specific for trimethylated H3K27, will be required to confirm that the regions of high FRET do correspond with regions of high H3K27 trimethylation. One confounding factor could be local differences in H2B abundance that cause increased or decreased amounts of intermolecular FRET.

### Prospects for the dual expression library screening strategy

With the successful identification of linker combinations that provide improved ratio changes, we have demonstrated that this dual expression library screening strategy, in its current form, is of practical utility for optimizing FRET-based biosensors designed to respond to post-translational modification. We expect that this system could be used to optimize a wide variety of biosensors for post-translation modification, provided that the a constitutively active enzyme with the activity of interest can be expressed in its functional form in *E. coli*. However, the current implementation of the screening system does have some drawbacks that will hopefully be addressed in future versions. One drawback of the current implementation is that, while the colony-based screen did allow us to identify a population of the best variants from a large library, it was not sufficient for identifying the single best variant. Accordingly, a secondary in vitro test was required for identification of the variants with the highest FRET changes. A second drawback was our reliance on manual spotting for the replication of bacterial colonies, which severely limited the throughput of the assay and increased the likelihood of human error. An improved method for library screening would involve the on-plate induction of enzyme expression. With this approach, images of the same plate, before and after enzyme induction, could be acquired and processed to identify the colonies with the largest changes in emission ratio. Our own efforts to achieve on-plate induction using the P_BAD _promoter and application of L-arabinose by spraying did not provide satisfactory results due to the heterogeneity of the application. We expect that this limitation could be overcome through the use of a promoter that could be induced at the same level across the whole plate. One potential solution is to use a cold-inducible promoter [26], which could presumably be induced at the same level in all colonies by simply changing the incubation conditions of the plate. However, it is unclear whether an alternate promoter could provide an equivalent level of repression to P_BAD _in the uninduced state.

With improvements in this library screening strategy, it should be possible to substantially increase the screening throughput. In the current implementation, we were limited to screening hundreds of variants and thus our screen is not exhaustive and has not necessarily yielded the optimal linker combination. In addition, we only investigated the effect of linker length on ratio change and did not attempt to adjust linker composition. We strongly suspect that further improvements in ratio change could be achieved by screening of larger number of variants and exploring both altered linker lengths and compositions [27,28]. We also suspect that biosensors with further improved ratio changes could be identified by altering the orientation of the donor and acceptor FPs by employing circularly permuted variants [29] or using 'sticky' FP variants [30].

## Conclusion

We have developed a method for high-throughput screening of biosensor libraries with many hundreds of different linker combinations. This approach should be applicable to the optimization of any genetically encoded biosensor for post-translational modification, provided the gene encoding the enzyme activity of interested can be functionally expressed in *E. coli*. We have demonstrated this technology by undertaking the optimization of a biosensor for detection of methylation of H3K27. Furthermore, we have shown that mammalian cells expressing this biosensor as a histone fusion are viable. Accordingly, we anticipate that H3K27-MetBio3 may facilitate future efforts to spatially resolve H3K27-trimethylation patterns in living cells. Furthermore, when compared to existing *in vitro *assays for histone methyltransferase activity [31-33], this homogenous biosensor-based assay is notable for requiring the least number of reagents and liquid handling steps. Accordingly, H3K27-MetBio3 may be particularly useful in automated high-throughput screening efforts aimed at the identification of H3K27 methyltransferase inhibitors that could serve as chemical probes [34] or as leads for the development of new cancer therapies [35].

## Methods

### General methods and materials

All primers and polynucleotides were purchased from Integrated DNA Technologies and their sequences are provided in Table S1, Additional file 2. Polymerase chain reaction (PCR) was performed using either *Taq *DNA polymerase (Invitrogen) or *Pfu *DNA polymerase (Fermentas) according to the provided manufacturers' protocols. Restriction endonucleases were purchased from New England Biolabs and used in the buffers recommended by the manufacturer. For routine analytical DNA digestion, FastDigest^® ^restriction endonucleases (Fermentas) were used. QIAquick Gel-Extraction kit (Qiagen) was used for DNA purification following agarose gel electrophoresis. DNA ligation was carried out using T4 DNA ligase (Invitrogen). *E. coli *strain ElectroMAX DH10B™ (Invitrogen) was used for routine plasmid propagation, library construction and screening, and recombinant protein production. Plasmid DNA for both bacterial transformation and mammalian cell transfection was isolated and purified using Fermentas GeneJET™-plasmid miniprep kit following the manufacturer's protocol. DNA sequencing reactions were performed using either DYEnamic™ ET (Amersham) or BigDye^® ^(Applied Biosciences) terminator cycle sequencing kits.

Bicinchoninic acid (BCA) protein assay (Pierce) was routinely used to measure protein concentration following the manufacturer's microplate format procedure in 96-well plates (Corning) which were read on a Safire2 microplate reader (Tecan). To determine the concentration of chimeric proteins that contained mTFP1 [18], absorbance at 462 nm was recorded on DU-800 spectrophotometer (Beckman-Coulter) and concentration was calculated using Beer-Lambert's law. To assess the purity of recombinant proteins, Glycine-SDS-PAGE or Tricine-SDS-PAGE - the choice of which was based on the estimated molecular weight of the protein of interest - was performed using 2 μg protein/lane [36] and PageRuler™ prestained protein ladder (Fermentas). Unless otherwise indicated, all chemicals and reagents were purchased from Fisher Scientific. All culture media contained ampicillin at a final concentrations of 0.04%.

### Construction of the first generation biosensor H3K27-MetBio1 and lib1

The gene encoding mTFP1 was used as the initial template for three rounds of successive PCRs using the primers combinations: 'Trugon1 Forward1 (Seq)' and 'Trugon1 Backward1 (Compl)'; 'Trugon2 Forward2 (Seq)' and 'Trugon2 Backward2 (Compl)'; and 'Trugon3 Forward3 (Seq)' and 'Trugon3 Backward3 (Compl).' The amplified product was digested with XhoI and EcoRI and ligated into pBAD/His B plasmid that was digested with the same enzymes. The resulting plasmid (referred to as 'Trugon-TFP') had the five unique restriction sites XhoI, XbaI, AvrII, PstI and BclI upstream of mTFP1 sequence; and BglII, EcoRI and HindIII downstream of it. A separate PCR was performed to amplify mTFP1 with 'TFP-XhoI-FD-SEQ' and 'TFP-XbaI-BK-CPL' primers. The PCR-product was digested with XhoI and XbaI enzymes and ligated into pBAD/His B based 'Trugon-TFP' that was digested with the same restriction enzymes. The resulting plasmid was digested with XbaI and PstI for use in a subsequent ligation reaction.

The potential binding domains were each subcloned into a pBAD/His B-derived plasmid that had the following nucleotide scheme: *AvrII*-protein domain-218 linker-*XbaI*-*PstI*. The '218 linker' is a previously reported sequence with good proteolytic stability [20]. Plasmids containing these protein domains were each digested with AvrII and PstI and the obtained inserts were ligated with the previously mentioned XbaI and PstI digested plasmid. The plasmid products were digested with XbaI and HindIII enzymes and used in subsequent ligation reactions. A nucleotide sequence encoding the histone-derived substrate sequence were added to the 5' end of mCitrine through two rounds of successive with the primers: '1-XbaI-H3K27-SEQ' and 'YFP-BK-CPL'; and '2-XbaI-H3K27-SEQ' and '2-YF-BK-CPL.' The obtained products were digested with XbaI and HindIII enzymes and ligated with the previously mentioned XbaI and HindIII digested plasmid. This procedure produced plasmids encoding the 7 distinct potential biosensor constructs.

### Construction of the dual expression plasmid (pUADE)

To construct pUADE, the two primers 'pGEX-For-TAC (Seq)' and 'pGEX-Back-TAC (Comp)' were used to amplify a 264 bp fragment that contained P*_tac _*from pGEX-6P-1 plasmid (GE life sciences) and to introduce *BamHI *and *NcoI *sites at that 5' and 3' ends, respectively, of the amplification product. The obtained DNA fragment was digested with BamHI and NcoI and was ligated into pET22b plasmid (Novagen) that had been digested with BglII and NcoI. Next, 'Forward-BAD-transfer' and 'Backward-BAD-transfer' were used to amplify the section of pBAD/His B extending from nucleotide 333 to 765 (original plasmid numbering) and to introduce *PciI *and *BglII *sites at the 5' and 3' ends respectively. The obtained DNA was digested with PciI and BglII and was ligated with the NcoI and BamHI digested pET22b plasmid from the previous step. The resulting plasmid was used as a template for a PCR using the primers 'POLY-Prmr1(pet)-Frwd' and 'POLY-Prmr1(pet)-Backwrd' to amplify the part of the plasmid that encoded the LacI, the modified promoter and its downstream multiple cloning site (MCS) and transcription terminator. Digestion of the resulting DNA fragment with PmlI provides the fragment designated as 'POLY-Prmr1(pet)'.

A dsDNA fragment with 5' and 3' overhangs compatible for ligation with XhoI and HindIII digested DNA respectively was obtained by slowly cooling a buffered solution of 'MCS-SEQUENCE' and 'MCS-COMPLEMENTARY' polynucleotides, at a concentration of 0.5 mM each, from 95°C to room temperature (RT) at a rate of -1°C every 3 min. The obtained dsDNA was ligated with pBAD/His B plasmid (Invitrogen) that had been digested with XhoI and HindIII. This modified pBAD plasmid was then digested with EcoRI and BglII and ligated with the a similarly digested DNA fragment created by PCR amplification of the vSET genes using 'vSET-EcoRI-Frwd (seq)' and 'vSET-BglII-Bckd (Compl).' The resulting plasmid, named 'New MCS-pBAD,' was digested with BsaAI and ligated with the 'POLY-Prmr1(pet)' fragment described above. This procedure provided the pUADE plasmid that had the vSET gene inserted in an MCS under P_BAD _and a second MCS under P*_tac_*. pBAD/His B vectors containing the aforementioned seven H3K27 methylation biosensors were pooled together in equimolar amounts and subsequently digested with XhoI and HindIII restriction enzymes. DNA electrophoresis followed by gel extraction were performed to purify the nucleotide inserts encoding the biosensors which were subsequently ligated into XhoI-HindIII restricted pUADE.

### Construction of lib2 and lib3 linker libraries

To facilitate cloning of linker libraries, we incorporated new restriction sites between the constituting parts of the H3K27-MetBio to have the following scheme: XhoI-mTFP1-*KpnI*-Cbx7-*SalI*-H3K27-*EagI*-mCitrine-HindIII. To introduce the new restriction sites (italicized) in the MCS downstream of P_Tac _in pUADE, a stepwise assembly of an initial construct with these new sites was performed followed by its introduction in pUADE. Briefly, double stranded DNA encoding the H3K27 substrate was obtained by slowly cooling a buffered solution that contained both coding polynucleotide strand 'SalI(RF2)-H3K27-Sense' and its reverse compliment 'EagI(RF3)-H3K27-Antisense' (at 0.5 μM each) from 95°C to 10°C at temperature drop rate of -1°C every 3 min. The product was purified using QIAquick Nucleotide Removal Kit after which it was digested with SalI and re-purified. The obtained product was ligated with SalI digested Cbx7 that had been previously amplified using 'FrWd-KpnI(RF1)-Cbx7' and '0-BkWd-SalI(RF2)-Cbx7' primers. After four hours of ligation, an aliquot of the reaction mixture was used as the template in a PCR reaction with the primers 'LigCbx7-H3K27-forward' and 'LigCbx7-H3K27-Backward'.

Purified product from the previous step was digested with EagI and ligated with similarly digested mCitrine that had been previously amplified using '0-FrWd-EagI(RF3)-YFP' and 'YFP-Primer 4' primers. The ligation reaction was allowed to proceed for four hours before an aliquot was removed and used as the template in a PCR with the 'FrWd-KpnI(RF1)-Cbx7' and 'YFP-Primer 4' primers. The product of this PCR reaction was a 3-part chimera encoding Cbx7-H3K27-mCitrine. This product was digested with KpnI and ligated with KpnI-digested mTFP1 that had been previously amplified using the 'TFP-Primer 1' and '0-BkWd-KpnI(RF1)-TFP' primers. The ligated construct was subsequently subjected to an amplification step using 'TFP-Primer 1' and 'YFP-Primer 4' primers to produce the full 4-part chimera encoding H3K27-MetBio with the new restriction sites. This PCR product was digested with XhoI and HindIII and ligated with the pUADE-vSET plasmid that had been digested with the same enzymes. This plasmid was then used for the construction of the linker lengths. Primers for mTFP1 were designed to incorporate an *XhoI *site at the 5' end and a *KpnI *site at the 3' end. For mTFP1, all deletions or extensions at the C-terminus are relative to the codon for residue 225 [18]. Primers for Cbx7 were designed to introduce *KpnI *and *SalI *at the 5' and 3' ends respectively. Similarly, primers for mCitrine introduced an *EagI *site at the 5' end and a stop codon followed by a *HindIII *site at the 3' end. PCR amplifications with each pair of primers were done individually, and the resulting PCR products pooled prior to digesting and ligation into pUADE. Following ligation, 3 μL of the reaction mixture were used to transform 60 μL of electrocompetent *E. coli *which were then added to 3 mL of fresh LB medium containing ampicillin in 10 mL culture tube. After 16 h at 37°C with shaking at 220 rpm, the culture was spun down and plasmid DNA was extracted. The obtained plasmid DNA was then digested with EagI and HindIII enzymes to be used in ligation reaction with mCitrine library. Ligation, transformation and plasmid DNA preparation proceeded as described above. Similarly, the KpnI and SalI digested Cbx7 library was ligated into the pUADE plasmid that contained mTFP1 and mCitrine libraries.

### Western blot procedure

*E. coli *was transformed with vSET in pBAD/His B and spread on plates that had been prepared with IPTG (1 mM) and various concentrations L-arabinose (Sigma) and D-glucose. Colonies were gently scraped off the plates and B-PER II (Pierce) was used to extract the soluble proteins from the cell paste according to the manufacturer's protocol. Protein concentration was then determined and 2 μg and 20 μg (in equal volumes) were subjected to Tricine-SDS-PAGE. Proteins were then electroblotted on PVDF membrane (Millipore) and tris-buffered saline pH 7.4 containing 0.1% Tween-20 and 5% non-fat milk was used to block the membrane overnight at 4°C. The membrane was washed, incubated with anti-His antibody conjugated with horseradish peroxidase (Roche), and rewashed. Locations of the 6 × His tagged proteins were then detected using ECL chemiluminescence substrate (Pierce) and BioMax light film (Kodak).

### Library screening procedure

The library of FRET-based biosensors in pUADE (with vSET) was used to transform *E. coli *and transformants were grown on LB-agar (0.04% ampicillin) in polystyrene Petri dishes. Sterilized toothpicks were used to stab individual colonies and then spot them on two LB-agar plates at the same relative position. One plate contained LB/agar supplemented with 0.04% ampicillin, 10 mM L-arabinose, 10 mM D-glucose and 1 mM IPTG and one plate contained 0.04% ampicillin, 20 mM D-glucose and 1 mM IPTG. Following overnight incubation at 37°C, fluorescence images were acquired using custom-made imaging system that has been previously described [21]. Custom macros were used to process the acquired images and create a spreadsheet where each row contained a colony identification number along with its donor and sensitized acceptor emission intensities from both plates. The spreadsheet was exported to Microsoft Excel where a Δratio values were calculated for each colony. Colonies that showed the highest ratio change were propagated and their plasmid DNA was extracted and sequenced.

### Protein purification

Unless otherwise indicated, pBAD/His B was the expression plasmid for recombinant protein production, with nucleotide sequences encoding proteins of interest inserted between the *XhoI *and *HindIII *sites downstream P*_ara _*and in-frame with the 5' 6 × His tag. Over-expression of the 6 × His-tagged proteins was achieved by L-arabinose (0.2%) induction of transformed *E. coli *cultured in 500 mL LB-Lennox media supplemented with ampicillin (0.04%). The culture was incubated for 22 hours at 37°C with shaking at 240 rpm. Bacterial cells were collected by centrifugation at 5000 rpm and resuspended in 30 mL lysis Buffer (50 mM sodium phosphate, 300 mM sodium chloride, 10 mM imidazole, pH 7.5). The suspended cells were lysed using French pressure cell press, debris was pelleted by centrifugation at 14000 rpm, and Ni-NTA resin (Qiagen) were added to the cleared cell lysate to capture the 6 × His tagged proteins. Protein purification proceeded according to the procedure provided in the Ni-NTA manual. To minimize proteolytic degradation, Complete Protease inhibitor cocktail tablets (Roche) were added to all buffers used at the manufacturer's recommended dose and all steps were performed at 4°C. Proteins were further subjected to gel-filtration to isolate full-length protein from partially proteolyzed forms on an ÄKTA™-design HPLC using a HiLoad™ 16/60 Superdex™ 75 pg XK column (GE Life Sciences). Chromatographic separation was monitored by absorbance at 280 nm, 462 nm and 513 nm.

### *In vitro *FRET-based assay for vSET activity

The *in vitro *FRET assay for vSET activity was performed in clear bottom 96-well microplates (Corning). Each reaction contained 200 μM SAM, 1 μM vSET, 1 μM FRET-based biosensor protein, and 2 mg/mL bovine serum albumin (BSA) in a buffered solution (50 mM sodium phosphate, 300 mM sodium chloride, pH 8.0). Excitation wavelength was set to 420 nm and fluorescence emission was collected at 494 nm and 526 nm. Measurements were collected every 2 minutes for 6 h using a microplate reader. The ratio of fluorescence intensity at 526 nm to that at 494 nm was calculated at each time point. Ratios calculated for control wells that lacked SAM were averaged and these values were used as the baseline ratios (R_min_) for each biosensor construct. Changes in FRET were calculated as ΔR/R _min _(%) and were plotted against time.

### Mammalian cell imaging of H3K27-MetBio3 fused to H2B

To construct a mammalian expression vector encoding a fusion of H2B and H3K27-MetBio3, the H2B gene was PCR amplified with primers H2B-Xho1-F and H2B-Bgl2-FP-R and the M3K27-MetBio3 gene was PCR amplified with primers H2B-Bgl2-FP-F and pBAD-R. Both the H2B (5' *XhoI *and 3' *BglII*) and H3K27-MetBio3 (5' *BglII *and 3' *HindIII*) PCR products were subjected to restriction digest followed by purification and sequential ligations with a modified pcDNA3.1(-) vector. Mouse embryonic fibroblast (3T3) cells were cultured in DMEM (Sigma) supplemented with 10% Fetal Bovine Serum (FBS) (Sigma) at 37°C. Cells in 35 mm imaging dishes were incubated with 1 ml Dulbecco's Modified Eagle Medium (DMEM) (FBS free) for 30 min and then transfected with 1 μg plasmid DNA that had been mixed with 2 μl Turbofect™ Protein Transfection Reagent (Fermentas) in 0.1 ml DMEM (FBS free). The culture media was changed back to DMEM with 10% FBS after 2.5 h incubation at 37°C. Images were acquired 24-48 h post-transfection with cells at room temperature in HEPES buffered Hanks' Balanced Salt Solution (HHBSS) with 25 mM HEPES, 2 g/L D-glucose, 490 μM MgCl_2 _and 450 μM MgSO_4_, containing no phenol red.

Imaging was carried on an inverted Nikon Eclipse Ti microscope equipped with a 75 W xenon lamp (OSRAM) with a 25% neutral density filter, a 60 × objective (NA = 1.4 oil), and a 16-bit 512SC QuantEM CCD (Photometrics). The NIS-Elements AR 3.2 software package (Nikon) was used for automated computer control and for quantitative image analysis. The filters used for the donor channel were: 455/10 nm excitation; 470 nm dichroic; and 495/30 nm emission. For the FRET channel the filters were: 455/10 nm excitation; 495 nm dichroic; and 545/30 nm emission. Ratio images were created by dividing the FRET image by the donor image, applying a pseudocolor look-up-table, and scaling each pixel intensity in the ratio image by the corresponding pixel intensity for the average of the donor and FRET channels.

## Competing interests

REC is listed as an inventor on a U.S. patent that covers mTFP1

## Authors' contributions

AI designed and optimized the colony-based screening system and performed the construction and screening of biosensor libraries. HY performed phage display experiments and discovered the Cbx7 and JMJD2A variants. YD performed mammalian cell FRET imaging. REC conceived the project, designed experiments, and wrote the manuscript. All authors have read and approved the final version of this manuscript.

## Supplementary Material

Additional file 1 **Engineering of double tudor domain and chromodomain variants with altered binding specificities**. A detailed description of a research effort that involved using phage display-based screening for the development of improved reagents for molecular recognition of trimethylated H3K27. This work led to the engineering of 3 protein domains (JMJD2A double tudor domain with D945K; JMJD2A double tudor domain with the D945R; and Cbx7 chromodomain with A71K) that were used in the creation of lib1. Click here for file

Additional file 2 **Table S1 Synthetic oligonucleotide sequences**. Synthetic oligonucleotide sequences used in this study. Click here for file

## References

[B1] Ibraheem A Campbell RE Designs and applications of fluorescent protein-based biosensors Curr Opin Chem Biol 2010 14 30 36 10.1016/j.cbpa.2009.09.033 19913453

[B2] Miyawaki A Llopis J Heim R McCaffery JM Adams JA Ikura M Tsien RY Fluorescent indicators for Ca2+ based on green fluorescent proteins and calmodulin Nature 1997 388 882 887 10.1038/42264 9278050

[B3] Campbell RE Fluorescent-Protein-Based Biosensors: Modulation of Energy Transfer as a Design Principle Anal Chem 2009 81 5972 5979 10.1021/ac802613w 19552419

[B4] Pham E Chiang J Li I Shum W Truong K A computational tool for designing FRET protein biosensors by rigid-body sampling of their conformational space Structure 2007 15 515 523 10.1016/j.str.2007.03.009 17502097

[B5] Deuschle K Okumoto S Fehr M Looger LL Kozhukh L Frommer WB Construction and optimization of a family of genetically encoded metabolite sensors by semirational protein engineering Protein Sci 2005 14 2304 2314 10.1110/ps.051508105 16131659 PMC2253473

[B6] Russwurm M Mullershausen F Friebe A Jäger R Russwurm C Koesling D Design of fluorescence resonance energy transfer (FRET)-based cGMP indicators: a systematic approach Biochem J 2007 407 69 77 10.1042/BJ20070348 17516914 PMC2267402

[B7] Hires SA Zhu Y Tsien RY Optical measurement of synaptic glutamate spillover and reuptake by linker optimized glutamate-sensitive fluorescent reporters Proc Natl Acad Sci USA 2008 105 4411 4416 10.1073/pnas.0712008105 18332427 PMC2393813

[B8] Piljić A de Diego I Wilmanns M Schultz C Rapid development of genetically encoded FRET reporters ACS Chem Biol 2011 6 685 691 10.1021/cb100402n 21506563

[B9] Lin CW Jao CY Ting AY Genetically encoded fluorescent reporters of histone methylation in living cells J Am Chem Soc 2004 126 5982 5983 10.1021/ja038854h 15137760

[B10] Fischle W Wang Y Jacobs SA Kim Y Allis CD Khorasanizadeh S Molecular basis for the discrimination of repressive methyl-lysine marks in histone H3 by Polycomb and HP1 chromodomains Genes Dev 2003 17 1870 1881 10.1101/gad.1110503 12897054 PMC196235

[B11] Min J Zhang Y Xu RM Structural basis for specific binding of Polycomb chromodomain to histone H3 methylated at Lys 27 Genes Dev 2003 17 1823 1828 10.1101/gad.269603 12897052 PMC196225

[B12] Manzur KL Farooq A Zeng L Plotnikova O Koch AW Sachchidanand Zhou MM A dimeric viral SET domain methyltransferase specific to Lys27 of histone H3 Nat Struct Biol 2003 10 187 196 10.1038/nsb898 12567185

[B13] Qian C Wang X Manzur K Sachchidanand Farooq A Zeng L Wang R Zhou MM Structural insights of the specificity and catalysis of a viral histone H3 lysine 27 methyltransferase J Mol Biol 2006 359 86 96 10.1016/j.jmb.2006.03.006 16603186

[B14] de Boer HA Comstock LJ Vasser M The tac promoter: a functional hybrid derived from the trp and lac promoters Proc Natl Acad Sci USA 1983 80 21 25 10.1073/pnas.80.1.21 6337371 PMC393301

[B15] Guzman LM Belin D Carson MJ Beckwith J Tight regulation, modulation, and high-level expression by vectors containing the arabinose PBAD promoter J Bacteriol 1995 177 4121 4130 7608087 10.1128/jb.177.14.4121-4130.1995 PMC177145

[B16] Daugherty PS Olsen MJ Iverson BL Georgiou G Development of an optimized expression system for the screening of antibody libraries displayed on the Escherichia coli surface Protein Eng 1999 12 613 621 10.1093/protein/12.7.613 10436088

[B17] Dong H Nilsson L Kurland CG Gratuitous overexpression of genes in Escherichia coli leads to growth inhibition and ribosome destruction J Bacteriol 1995 177 1497 1504 7883706 10.1128/jb.177.6.1497-1504.1995 PMC176765

[B18] Ai H Henderson JN Remington SJ Campbell RE Directed evolution of a monomeric, bright and photostable version of Clavularia cyan fluorescent protein: structural characterization and applications in fluorescence imaging Biochem J 2006 400 531 540 10.1042/BJ20060874 16859491 PMC1698604

[B19] Bernstein E Duncan EM Masui O Gil J Heard E Allis CD Mouse polycomb proteins bind differentially to methylated histone H3 and RNA and are enriched in facultative heterochromatin Mol Cell Biol 2006 26 2560 2569 10.1128/MCB.26.7.2560-2569.2006 16537902 PMC1430336

[B20] Whitlow M Bell BA Feng SL Filpula D Hardman KD Hubert SL Rollence ML Wood JF Schott ME Milenic DE An improved linker for single-chain Fv with reduced aggregation and enhanced proteolytic stability Protein Eng 1993 6 989 995 10.1093/protein/6.8.989 8309948

[B21] Cheng Z Campbell RE Assessing the structural stability of designed beta-hairpin peptides in the cytoplasm of live cells ChemBioChem 2006 7 1147 1150 10.1002/cbic.200500540 16810655

[B22] Kim J Daniel J Espejo A Lake A Krishna M Xia L Zhang Y Bedford MT Tudor, MBT and chromo domains gauge the degree of lysine methylation EMBO Rep 2006 7 397 403 16415788 10.1038/sj.embor.7400625 PMC1456902

[B23] Kaustov L Ouyang H Amaya M Lemak A Nady N Duan S Wasney GA Li Z Vedadi M Schapira M Min J Arrowsmith CH Recognition and specificity determinants of the human cbx chromodomains J Biol Chem 2011 286 521 529 10.1074/jbc.M110.191411 21047797 PMC3013012

[B24] Peters AH Kubicek S Mechtler K O'Sullivan RJ Derijck AA Perez-Burgos L Kohlmaier A Opravil S Tachibana M Shinkai Y Martens JH Jenuwein T Partitioning and plasticity of repressive histone methylation states in mammalian chromatin Mol Cell 2003 12 1577 1589 10.1016/S1097-2765(03)00477-5 14690609

[B25] De Santa F Totaro MG Prosperini E Notarbartolo S Testa G Natoli G The histone H3 lysine-27 demethylase Jmjd3 links inflammation to inhibition of polycomb-mediated gene silencing Cell 2007 130 1083 1094 10.1016/j.cell.2007.08.019 17825402

[B26] Baneyx F Mujacic M Cold-inducible promoters for heterologous protein expression Methods Mol Biol 2003 205 1 18 12491876 10.1385/1-59259-301-1:01

[B27] Lissandron V Terrin A Collini M D'alfonso L Chirico G Pantano S Zaccolo M Improvement of a FRET-based indicator for cAMP by linker design and stabilization of donor-acceptor interaction J Mol Biol 2005 354 546 555 10.1016/j.jmb.2005.09.089 16257413

[B28] Ha JS Song JJ Lee YM Kim SJ Sohn JH Shin CS Lee SG Design and application of highly responsive fluorescence resonance energy transfer biosensors for detection of sugar in living Saccharomyces cerevisiae cells Appl Environ Microbiol 2007 73 7408 7414 10.1128/AEM.01080-07 17890334 PMC2168232

[B29] Nagai T Yamada S Tominaga T Ichikawa M Miyawaki A Expanded dynamic range of fluorescent indicators for Ca2+ by circularly permuted yellow fluorescent proteins Proc Natl Acad Sci USA 2004 101 10554 10559 10.1073/pnas.0400417101 15247428 PMC490022

[B30] Kotera I Iwasaki T Imamura H Noji H Nagai T Reversible dimerization of Aequorea victoria fluorescent proteins increases the dynamic range of FRET-based indicators ACS Chem Biol 2010 5 215 222 10.1021/cb900263z 20047338

[B31] Collazo E Couture JF Bulfer S Trievel RC A coupled fluorescent assay for histone methyltransferases Anal Biochem 2005 342 86 92 10.1016/j.ab.2005.04.007 15958184

[B32] Gowher H Zhang X Cheng X Jeltsch A Avidin plate assay system for enzymatic characterization of a histone lysine methyltransferase Anal Biochem 2005 342 287 291 10.1016/j.ab.2005.04.028 15935324 PMC2696273

[B33] Rathert P Cheng X Jeltsch A Continuous enzymatic assay for histone lysine methyltransferases Biotechniques 2007 43 602, 604, 606 passim 10.2144/000112623 PMC2703000 18072589

[B34] Cole PA Chemical probes for histone-modifying enzymes Nat Chem Biol 2008 4 590 597 18800048 10.1038/nchembio.111 PMC2908280

[B35] Simon JA Lange CA Roles of the EZH2 histone methyltransferase in cancer epigenetics Mutat Res 2008 647 21 29 10.1016/j.mrfmmm.2008.07.010 18723033

[B36] Schägger H Tricine-SDS-PAGE Nat Protoc 2006 1 16 22 10.1038/nprot.2006.4 17406207

[B37] Griesbeck O Baird GS Campbell RE Zacharias DA Tsien RY Reducing the environmental sensitivity of yellow fluorescent protein. Mechanism and applications J Biol Chem 2001 276 29188 29194 10.1074/jbc.M102815200 11387331

