# Supplementary material for: A bacteria colony-based screen for optimal linker combinations in genetically encoded biosensors
Source: BMC Biotechnol. 2011 Nov 10;11:105. doi: 10.1186/1472-6750-11-105 (PMC3225322; doi:10.1186/1472-6750-11-105)
Supplement: Additional file 1 — Engineering of double tudor domain and chromodomain variants with altered binding specificities. A detailed description of a research effort that involved using phage display-based screening for the development of improved reagents for molecular recognition of trimethylated H3K27. This work led to the engineering of 3 protein domains (JMJD2A double tudor domain with D945K; JMJD2A double tudor domain with the D945R; and Cbx7 chromodomain with A71K) that were used in the creation of lib1. [file 1472-6750-11-105-S1.PDF]

Additional file 1:

Engineering of double tudor domain and chromodomain variants with altered binding specificities

Hongkin Yap[1,2] and Robert E. Campbell*[1]

[1]Department of Chemistry, University of Alberta, Edmonton, Alberta T6G 2G2, Canada and

[2]Present address: The Hong Kong Polytechnic University, Hung Hom, Kowloon, Hong Kong, People's Republic of China

Email: HY - yaphongkin@gmail.com; REC - robert.e.campbell@ualberta.ca

* Corresponding author

Background

The first library we constructed and screened in this work (lib1) was composed of potential H3K27-trimethylation biosensors containing a variety of different binding domains. Among the binding domains included in this library were three engineered variants that have been developed in our lab and not previously reported elsewhere: JMJD2A double tudor domain [1,2] with D945K; JMJD2A double tudor domain with the D945R; and Cbx7 chromodomain [3] with A71K. These variants are the result of a research project aimed at using phage display-based screening for the development of improved reagents for molecular recognition of trimethylated H3K27. However, due to the relatively modest gains in specificity in these engineered variants, this work was not pursued further. This document describes the identification of these variants.

Results and Discussion

Phage display and panning of libraries based on JMJD2A double tudor domain

The JMJD2A double tudor domain has been reported to bind to trimethylated H3K4 ($H3K4Me_3$), trimethylated H3K9 ($H3K9Me_3$), dimethylated H4K20 ($H4K20Me_2$), and trimethylated H4K20 ($H4K20Me_3$) [2]. Based on this profile, it appeared to us that this

domain has relatively good specificity for the trimethylated state of the lysine side chain, and relative poor specificity for the peptide sequence. We speculated that it might be possible to engineer variants that retained the good specificity for the trimethylated state with improved specificity for the peptide sequence surrounding H3K27. Based on the published structure of the JMJD2A double tudor domain [1], we created a series of phage display libraries in which individual residues were subject to saturation mutagenesis. In each library, one of seven sites that interact with the bound histone tail were selected for saturation mutagenesis. The sites were F927, G935-S936, F937-S938, D939-N940, L941-Y942, E944-D945 and T968 (Figure 1). A saturation library was constructed for each one of these sites, and then all libraries were pooled prior to phage display based screening.

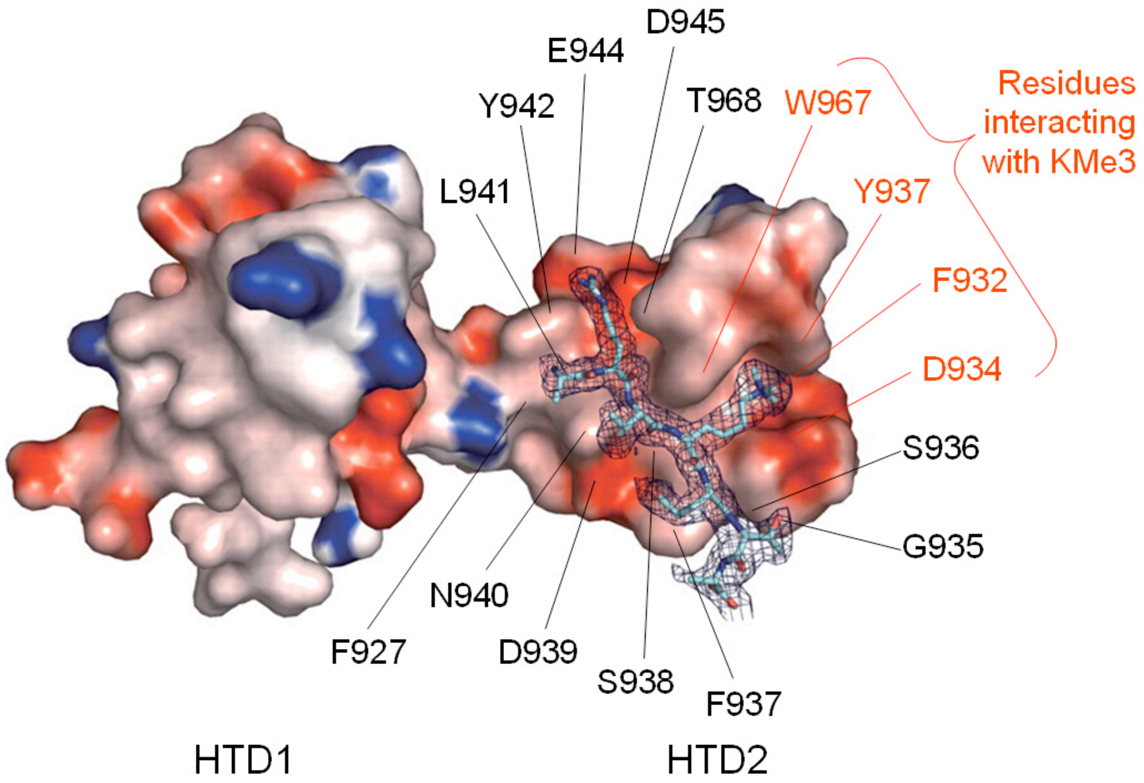

Figure 1 Crystal structure of the JMJD2A double tudor domain (PDB ID 2GFA) [1]**.** Labeled amino acid residues were selected for random mutagenesis to construct libraries for phage display screening.

After 3 rounds of panning the pooled libraries against immobilized H3K27Me$_3$, clones with

substitutions at position 944 and 945 was found to show the greatest increase in specificity towards H3K27Me$_3$. Further investigation revealed that substitutions at position 945 (specifically D945 to V, A, T, H, or R), were solely responsible for the altered specificities of these domains. Each of these variants was expressed on phage and a phage-binding assay was performed. In this assay, beads were incubated with phage and then washed thoroughly before phage was eluted. The eluted phage was used to infect *E. coli*, a portion of which was then plated on solid media. A higher the number of resulting colonies indicates a higher affinity of the protein domain for the immobilized peptide (Table 1).

The phage binding assay revealed that the D945K variant gave the greatest degree of discrimination (approximately 100-fold) between binding H3K27Me$_3$ (or H3K9Me$_3$) versus H3K4Me$_3$ (or H4K20Me$_3$). Disappointingly, there is no apparent specificity with respect to binding of H3K27Me$_3$ versus H3K9Me$_3$.

Table 1: Phage binding assay results of JMJD2A variants

Peptide	Normalized colony number[a]						
	Wild-type	D945V	D945A	D945T	D945H	D945R	D945K
H3K4Me$_3$	4.13±0.12	0.93±0.09	1.04±0.11	0.88±0.13	0.12±0.02	0.09±0.01	0.01±0.01
H3K9Me$_3$	0.98±0.05	0.97±0.02	0.98±0.03	0.98±0.05	0.99±0.04	1.01±0.03	0.95±0.02
H3K27Me$_3$	1[b]	1	1	1	1	1	1
H4K20Me$_3$	6.35±0.09	1.05±0.23	0.96±0.08	1.03±0.14	0.11±0.01	0.08±0.02	0.01±0.01

[a] Averages of four independent experiments.

[b] Colony number for H3K27Me$_3$ was normalized to 1.

Phage display and panning of libraries based on Cbx7 chromodomain.

In an attempt to improve the binding specificity of H3K27Me$_3$ versus H3K9Me$_3$ (and other histone-derived peptides), we also explored the use of the Cbx7 chromodomain as a template. This chromodomain has been reported to bind H3K27Me$_3$ (K_d = 22 ± 5 μM) and H3K9Me$_3$ (K_d = 12±3 μM) with similar affinities and with good selectivity relative to H3K4Me$_3$ and H4K20Me$_3$ (K_d >500 μM) [3].

As with the JMJD2A double tudor domain, we constructed and panned phage display libraries

of Cbx7 variants in order to identify clones with improved specificity for H3K27Me$_3$. Based on inspection of available structures of chromodomains with bound peptides [4], five sites proposed to interact with the bound histone tail were selected for the introduction of random mutations. The five sites were V25, A27, K48, L64 and A71. After 3 rounds of panning the pooled libraries, six mutants with possible higher binding affinity for H3K27Me$_3$ versus H3K9Me$_3$ were identified: V25S, V25R, K48R, L64G, L64S and A71K. Individual variants were expressed on phage and phage-binding assays were performed as described above (Table 2). This phage binding assay revealed that only the A71K substitution had a modest improvement in specificity for H3K27Me$_3$ versus H3K9Me$_3$.

Table 2: Phage binding assay results for Cbx7 variants

Peptide	Normalized colony number[a]						
	Wild-type	V25S	V25R	K48R	L64G	L64S	A71K
H3K9Me$_3$	1.06±0.03	0.95±0.03	1.15±0.01	1.03±0.02	0.95±0.11	0.88±0.06	0.23±0.02
H3K27Me$_3$	1[b]	1	1	1	1	1	1

[a] Averages of four independent experiments.

[b] Colony number for H3K27Me$_3$ was normalized to 1.

Conclusion

We have identified variants of the JMJD2A double tudor domain and the Cbx7 chromodomain with altered specificities for various histone H3-derived peptides that contain methylated lysines. Specifically, we have found that the JMJD2A double tudor domain D945K variant exhibits preferential binding to H3K27Me$_3$ and H3K9Me$_3$ over H3K4Me$_3$ and H4K20Me$_3$. Notably, the wild-type domain exhibits the opposite binding preference. We have also identified the Cbx7 chromodomain A71K variant that has a modestly improved preference for binding to H3K27Me$_3$ over H3K9Me$_3$.

Methods

General Methods

All reagents and procedures are identical to those described in the body of the manuscript

unless otherwise noted. The sequences of all primers used in this work are provided in Table S1, Additional file 2. Peptides were purchased from Pepmetric Technologies (Richmond, Canada).

Modification of pCANTAB5E to create pCANMOD

Two primers were designed to modify the plasmid pCANTAB5E (Amersham Biosciences):

PCAN-F which contains a *NotI* site, a *XbaI* site and an amber stop codon; and PCAN-R which contains a *NotI* site and a *BglII* site. A 50 µL PCR reaction mixture contained 20 mM Tris-HCl (pH 8.8), 10 mM KCl, 10 mM $(NH_4)_2SO_4$, 2 mM $MgSO_4$, 0.1% Triton X-100, 200 µM each dNTP, 10 pmol *PCAN-F*, 10 pmol *PCAN-R*, 0.5 µg pCANTAB5E and 1.25 U *Pfu* polymerase. A thermal cycling program was set to amplify the plasmid: (1) 95 °C: 3 min; (2) 35 cycles of 94 °C: 0.5 min, 50 °C: 0.5 min and 72 °C: 7 min; (3) 72 °C: 10 min. DpnI was added to the mixture after PCR and incubated at 37 °C for 1 hour. The PCR product was then purified from agarose gel and digested with NotI. After purification, the digested product was ligated and used to transform competent *E. coli*. The resulting plasmid was designated pCANMOD (Figure 2).

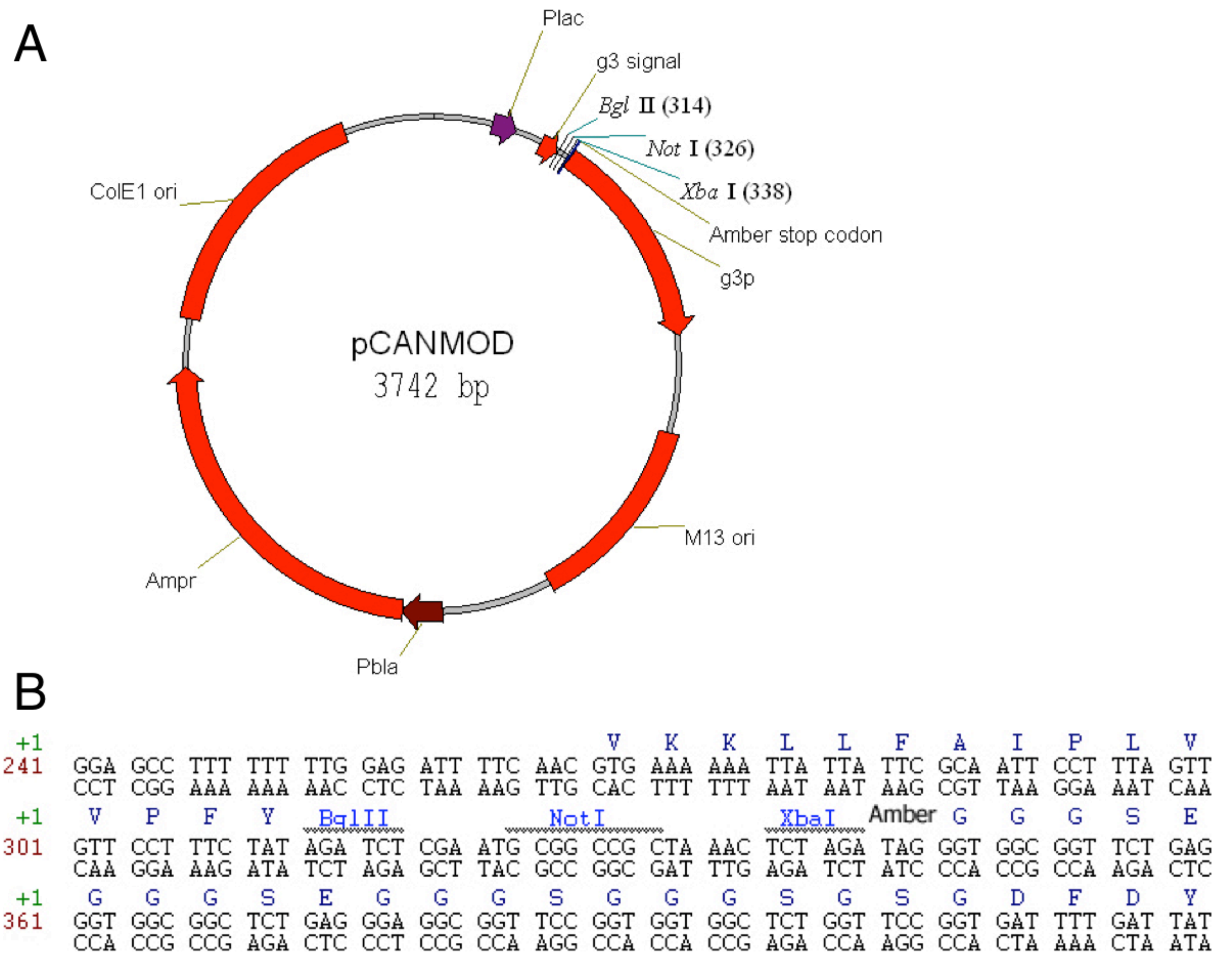

Figure 2 Plasmid used for phage display. (A) Map of pCANMOD. (B) Sequence of pCANMOD cloning site.

Cloning of the JMJD2A double tudor domain gene for phage display

The template cDNA encoding the JMJD2A double tudor domain [2] was subcloned into pCANMOD. Primers designed for amplification of the gene were JM-F which contained a *BglII* site, and JM-R which contained a *XbaI* site. A 50 µL PCR reaction mixture contained 20 mM Tris-HCl (pH 8.8), 10 mM KCl, 10 mM $(NH_4)_2SO_4$, 2 mM $MgSO_4$, 0.1% Triton X-100, 200 µM each dNTP, 10 pmol JM-F, 10 pmol JM-R, 0.5 µg PCR product of tudor domain and 1.25 U *Pfu* polymerase. A thermal cycling program was set to amplify the gene: (1) 95 °C: 3 min; (2) 35 cycles of 94 °C: 0.5 min, 50 °C: 0.5 min and 72 °C: 1 min; (3) 72 °C: 10 min. The PCR product was purified from agarose gel and digested with BglII and XbaI. After purification, the digested product was ligated with a BglII and XbaI predigested pCANMOD and transformed into *E. coli* TG1. The sequence of pCAN-JMJD was confirmed by DNA

sequencing.

Construction of JMJD2A tudor domain libraries

Mutagenesis to create JMJD2A tudor domain libraries was achieved by performing two rounds of PCR for each position to be targeted. The primers (sequences provided in Table S1, Additional file 2) to target a particular position follow the naming scheme [site]-F or [site]-R for forward and reverse primers, respectively. For example, the primers to target position 927 are labeled as F927-F and F927-R. In the first round one PCR was carried out using [site]-F, JM-R, and pCAN-JMJD as the template. A second PCR was carried out using and [site]-R, JM-F, and pCAN-JMJD as the template. The PCR products of both PCR reactions were purified by Qiagen gel extraction kit and eluted in 50 μL ddH_2O. In the second round PCR, the 50 μL PCR reaction mixture contained 20 mM Tris-HCl (pH 8.8), 10 mM KCl, 10 mM $(NH_4)_2SO_4$, 2 mM $MgSO_4$, 0.1% Triton X-100, 200 μM each dNTP, 10 pmol JM-F, 10 pmol JM-R, 3 μL of each purified PCR product from tube A and tube B, and 1.25 U *Pfu* polymerase. A thermal cycling program was set to amplify the mutated gene: (1) 95 °C: 3 min; (2) 35 cycles of 94 °C: 0.5 min, 50 °C: 0.5 min and 72 °C: 1 min; (3) 72 °C: 10 min. The PCR product was purified from agarose gel and digested with BglII and XbaI. After purification, the digested product was ligated with a BglII and XbaI predigested pCANMOD and transformed into *E. coli* TG1.

Preparation of helper phage stock

A single *E. coli* XL1-blue colony was picked from a fresh 2×TY/tet plate into 2 mL of 2×YT/tet media and incubated for 6-8 hours at 37 °C with shaking at 200 rpm. M13K07 helper phage was added to a final concentration of 10^{10} phage/mL and incubated for 30 min at 37 °C with shaking. The culture was transferred to 250 mL of 2×YT/kan media in a 1 L baffled flask (M13K07 carries a kanamycin resistance marker) and incubated overnight at 37 °C with shaking at 200 rpm. The overnight culture of phage was centrifuged for 10 min at 10K rpm in 4 °C. The supernatant was transferred into a fresh centrifuge tube and a 20% volume of PEG/NaCl solution (200 g/L PEG-8000, 2.5 M NaCl) was added and incubated for 10 min at RT to precipitate the phage. The solution was then centrifuged for 10 min at 10K rpm in 4 °C and the supernatant was carefully decanted. The phage pellet was resuspended in 5% volume of PBS. The insoluble matter was pelleted by centrifuging for 5 min at 15K rpm in 4 °C. The supernatant was then transferred to a clean tube and PBS added to a final

concentration of 10^{13} phage/mL ($OD_{268} = 1.0$ for a solution containing 5×10^{12} phage/mL). The protocol yield at least 25 mL of M13K07 helper phage stock. The phage solution was sterilized by passing through 0.45 μm membrane filter before storage at 4 °C for short term or -70 °C for long term.

Preparation of phage display libraries of double tudor domain variants

Plasmid pCAN-JMJD, or the mixture of plasmids with random mutations at 7 different sites, was used to transform *E. coli* TG1 competent cells. One milliliter LB medium was immediately added to the transformed cells. Following incubation for 1 hour at 37 °C with shaking at 250 rpm, 10 μL of the culture was plated and incubated overnight at 37 °C. The other 100 μL of transformed cells was transferred into LBK medium and grown to prepare a frozen stock. Then 9 mL of 2×YT medium was added into the remaining transformed cells or frozen stock cells and incubated for 1 hour at 37 °C with shaking at 250 rpm. After incubation, 4×10^{10} phage was added to the cells suspension and kanamycin was added to a final concentration of 10 mg/mL. The culture was incubated at 37 °C with shaking at 250 rpm overnight. After overnight growth, *E. coli* TG1 cells were pelleted by centrifugation, and the phage were precipitated from the supernatant with 20% volume of PEG/NaCl solution. The phage were pelleted by centrifugation and resuspended in 1% of the original culture volume of PBS.

Preparation of biotinylated histone-derived peptides

All peptide sequences are provided in Table 3. To deprotect the Cys(StBu)-OH group in the peptides, ~5 mg of peptide was weighed and a 10-fold molar excess of Tri(2-chloroethyl) phosphate (TCEP) was mixed in ~ 2 mL of 20 mM sodium phosphate buffer (pH 7.0). The pH was estimated by pH paper and adjusted to pH 7.0 by addition of 2 M NaOH. The mixture was incubated at RT for 2 hours with gentle mixing and dialyzed in 2 L of 20 mM sodium phosphate buffer (pH 7.0) by using Spectra/Por® CE dialysis tubing, 500 MWCO (Spectrum Laboratories) to remove the cleaved StBu. Dialysis was done in cold room overnight. A 10-fold molar excess of TCEP was added to the dialyzed deprotected peptide and the pH was adjusted to 7.0 before conjugation. The mixture was then mixed with equimolar amount of EZ-Link maleimide PEO_2-Biotin (PIERCE) that had been freshly prepared as a 20 mM solution in PBS. The reaction was incubated at RT for 2 hours with gentle mixing and dialyzed at 4 °C overnight in 2 L of 20 mM sodium phosphate buffer (pH 7.0) by using

Spectra/Por® CE dialysis tubing, 500 MWCO. The conjugated product was confirmed by mass spectrum and the concentration of peptide was determined spectrophotometrically, assuming an extinction coefficient of 1280 $M^{-1}\cdot cm^{-1}$ at 280 nm due to the presence of a single tyrosine residue.

Table 3: Sequences of histone derived peptides used in this work

Peptide	Sequence
$H3K9Me_3$	GTKQTAR[Kme3]STGGGY[CtBu]
$H3K9Me_2$	GTKQTAR[Kme2]STGGGY[CtBu]
$H3K9Me_1$	GTKQTAR[Kme1]STGGGYC
H3K9	GTKQTARKSTGGGYC
$H3K27Me_3$	GATKAAR[Kme3]SAPAGY[CtBu]
$H3K27Me_2$	GATKAAR[Kme2]SAPAGY[CtBu]
$H3K27Me_1$	GATKAAR[Kme1]SAPAGYC
H3K27	GATKAARKSAPAGYC
$H3K4Me_3$	ART[Kme3]QTARGYC
$H3K4Me_2$	ART[Kme2]QTARGYC
$H3K4Me_1$	ART[Kme1]QTARGYC
H3K4	ARTKQTARGYC
$H3K36Me_3$	GPATGGV[Kme3]KPHRGYC
$H3K36Me_2$	GPATGGV[Kme2]KPHRGYC
$H3K36Me_1$	GPATGGV[Kme1]KPHRGYC
H3K36	GPATGGVKKPHRGYC
$H4K20Me_3$	GGAKRHR[Kme3]VLRDGYC
$H4K20Me_2$	GGAKRHR[Kme2]VLRDGYC
$H4K20Me_1$	GGAKRHR[Kme1]VLRDGYC
H4K20	GGAKRHRKVLRDGYC

Immobilization of biotinylated peptide on magnetic beads

To 90 μL of PBS in a 1.5 mL microcentrifuge tube was added 10 μL of Dynabeads® M-280 Streptavidin (Invitrogen, supplied in 10 mg/mL). Following resuspension, the tube was placed

adjacent to a strong magnet for 1-2 min. The supernatant was removed with a pipette and the beads were gently resuspended 100 μL of PBS. The washing step was repeated four times, and the beads were finally resuspended at a concentration of 10-50 mg/mL. Biotinylated peptide (20 pmol) was added to the resuspended beads and incubated at RT for 20 min with gentle mixing. The beads were washed four times and resuspended in 10 μL of PBS with 0.01% Tween20. The beads were immediately used in phage display panning.

Panning of phage display libraries

Approximately 20-2000 pmol of non-biotinylated free peptides were added together with 10^{11} phage and magnetic beads to which biotinylated peptides had been immobilized in a final volume of 20 μL of PBS with 0.005% Tween20. The mixture was incubated at RT and mixed gently in 5 min interval. The beads were then washed with washing buffer (PBS, 0.05% Tween20) nine times, with an additional washing step using PBS as washing buffer. The bound phage were eluted by adding 20 μL of 50 μM non-biotinylated free peptide with the same sequence as the immobilized peptide and incubating for 30 min at RT with gentle mixing at 5 min time intervals. The tube was placed on a magnet and the supernatant with eluted phage was transferred to a new tube for infection step. The eluent was added to 2 mL of log-phase TG1 cells (in 2×TY) and incubated at 37 °C with shaking at 250 rpm for 30 min. From this culture, 10 μL of cell was withdrawn and undergone serial dilution and spread on LB/amp plate to check number of phage obtained. Ampicillin and 4×10^9 plaque forming units of M13KO7 were added and incubated in same condition for 30 more minutes. The 2 mL culture was then transferred to 50 mL 2×TY/kan/amp medium and incubated at 37 °C with shaking at 250 rpm overnight. Phage was precipitated and used in the next round of panning.

Construction of the gene encoding the Cbx7 chromodomain

Eight primers were designed for use in construction of the gene of Cbx7 chromodomain by overlap extension PCR (C71-F through C78-R in Table S1, Additional file 2). Two rounds of PCR were performed to construct the gene. In the first round, a 50 μL PCR reaction mixture contained 20 mM Tris-HCl (pH 8.8), 10 mM KCl, 10 mM $(NH_4)_2SO_4$, 2 mM $MgSO_4$, 0.1% Triton X-100, 200 μM each dNTP, 1 pmol of each primers and 1.25 U *Pfu* polymerase. A thermal cycling program was set to amplify the gene: (1) 95 °C: 1 min; (2) 35 cycles of 94 °C: 0.5 min, 50 °C: 0.5 min and 72 °C: 0.5 min. In the second round, a 50 μL PCR reaction mixture contained 20 mM Tris-HCl (pH 8.8), 10 mM KCl, 10 mM $(NH_4)_2SO_4$, 2 mM $MgSO_4$,

0.1% Triton X-100, 200 μM each dNTP, 10 pmol C7Bgl-F, 10 pmol C7Xba-R, 1 μL PCR reaction from the first round and 1.25 U *Pfu* polymerase. A thermal cycling program was set to amplify the gene: (1) 95 °C: 3 min; (2) 35 cycles of 94 °C: 0.5 min, 50 °C: 0.5 min and 72 °C: 1 min; (3) 72 °C: 10 min. The PCR product was purified by agarose gel electrophoresis and digested with BglII and XbaI. After purification, the digested product was ligated with a BglII and XbaI predigested pCANMOD and transformed into *E. coli* TG1. The sequence of pCAN-Cbx7 was confirmed by DNA sequencing.

Construction of mutants of Cbx7 chromodomain

The protocol for mutagenesis was essentially identical to that described above for the JMJD2A double tudor domain. Template pCAN-Cbx7 was used in place of pCAN-JMJD and primers C7Xba-R and C7Bgl-F were used in place of JM-R and JM-F, respectively.

Phage display and panning

The procedures for phage display and panning of Cbx7 mutant libraries are identical to those described above for the JMJD2A double tudor domain.

References

1. Huang Y, Fang J, Bedford MT, Zhang Y, Xu RM: **Recognition of histone H3 lysine-4 methylation by the double tudor domain of JMJD2A.** *Science* 2006, **312**:748-751.

2. Kim J, Daniel J, Espejo A, Lake A, Krishna M, Xia L, Zhang Y, Bedford MT: **Tudor, MBT and chromo domains gauge the degree of lysine methylation.** *EMBO Rep* 2006, **7**:397-403.

3. Bernstein E, Duncan EM, Masui O, Gil J, Heard E, Allis CD: **Mouse polycomb proteins bind differentially to methylated histone H3 and RNA and are enriched in facultative heterochromatin.** *Mol Cell Biol* 2006, **26**:2560-2569.

4. Fischle W, Wang Y, Jacobs SA, Kim Y, Allis CD, Khorasanizadeh S: **Molecular basis for the discrimination of repressive methyl-lysine marks in histone H3 by Polycomb and HP1 chromodomains.** *Genes Dev* 2003, **17**:1870-1881.
