# Supplementary material for: A bacteria colony-based screen for optimal linker combinations in genetically encoded biosensors
Source: BMC Biotechnol. 2011 Nov 10;11:105. doi: 10.1186/1472-6750-11-105 (PMC3225322; doi:10.1186/1472-6750-11-105)
Supplement: Additional file 2 — Table S1 Synthetic oligonucleotide sequences. Synthetic oligonucleotide sequences used in this study. [file 1472-6750-11-105-S2.PDF]

Additional file 2:

Table S1 Synthetic oligonucleotide sequences

Primer	Sequence (5' to 3')
Trugon1 Forward1 (Seq)	CTGCAGACTGATCATATGGTGAGCAAGGGCGAGGAG
Trugon1 Backward1 (Compl)	TTTTTGTTCCAGATCTAGCTTGTACAGCTCGTCCATGCCGTC
Trugon2 Forward2 (Seq)	AGAAATTACCCTAGGCTGCAGACTGATCATATGGTGAG
Trugon2 Backward2 (Compl)	ATCCTCTTCTGAGATGAGTTTTTGTTCCAGATCTAGCTTGTACAG
Trugon3 Forward3 (Seq)	AATATCTCGAGCTCTAGAAATTACCCTAGGCTGCAGA
Trugon3 Backward3 (Compl)	TACATGGAATTCTTACAGATCCTCTTCTGAGATGAGTTTTTGTTC
TFP-XhoI-FD-SEQ	CTATATGACCTCGAGCATGGTGAGCAAGGGCGAGGAGCTGTT
TFP-XbaI-BK-CPL	ACGCCAAAACAGCCATCTAGACTTGTACAGCTCGTCCATGCCGAGAGTGAT
1-XbaI-H3K9-YFP-SEQ	GCTAGAAAGTCTACAGGAGGAAAGGCTATGGTGAGCAAGGGCGAGGAGCTGTT
2-XbaI-H3K9-YFP-SEQ	CGTGACCATTATGCCTCTAGAATGGCCCGTACTAAGCAGACTGCTAGAAAGTCTACAGGAGGAAAG
1-XbaI-H3K27-SEQ	AGAAAGTCAGCACCTGCAACGGGAATGGTGAGCAAGGGCGAGGAGCTGTT
2-XbaI-H3K27-SEQ	CATAATCATTATGTATCTAGACTGGCCACCAAGGCGGCCAGAAAGTCAGCACCTGCAACG
YF-BK-CPL	ACGCCAAAACAGCCAAGCTTCTTACTTGTACAGCTCGTCCATGCCGAGAGTGAT
2-YF-BK-CPL	CCCGCTCTCCACGGAACGCCAAAACAGCCAAGCTT
pGEX-For-TAC (Seq)	CTATAAAGGATCCTTATCGACTGCACGGTGCACCAATGCTTC
pGEX-Back-TAC (Comp)	GATTTTATCCATGGTCATGAATACTGTTTCCTGTGTGAAATTGTTATCCGCTCAC
Forward-BAD-transfer	CTATAAAACATGTTACATCATCATCATCATCACGGTATGGCTAGCATGACT
Backward-BAD-transfer	CATTAAACATAGATCTCACCGACAAACAACAGATAAAACGAAAGGCCCAG
POLY-Prmr1(pet)-Frwrd	GTATTTCTAAAACACGTGATCCGGATATAGTTCCTCCTTTCAGCAAAAAACCCCTCAAGACCCGTTTAGAG G
POLY-Prmr1(pet)-Bckwrd	CGTCATTATACACGTGATAGTCATGCCCCGCGCCCACCGGAAGGAGCTGACT
MCS-SEQUENCE	TCGACCGAATTCTCCCCATTGGGTGGATATGGTGTATTTGCGAGAAAATCTTTCGAGAAGGGAGAACTTGT TGAAGAATGTTTGTGTATAGTGCGCCATAATGATGATTGGGGGACCGCCCTTGAAGATTATTTGTTTTCGA GAAAGAATATGTCTGCAATGGCTCTTGGTTTTGGTGCAATTTTTAACCATAGATCTGC
MCS-COMPLEMENTARY	AGCTGCAGATCTATGGTTAAAAATTGCACCAAAACCAAGAGCCATTGCAGACATATTCTTTCTCGAAAACA AATAATCTTCAAGGGCGGTCCCCCAATCATCATTATGGCGCACTATACACAAACATTCTTCAACAAGTTCT CCCTTCTCGAAAGATTTTCTCGCAAATACACCATATCCACCCAATGGGGAGAATTCGG
vSET-EcoRI-Frwd (seq)	GATATATTGAATTCATGTTTAATGACAGAGTCATCGTGAAAAAATCCCCATTG
vSET-BglII-Bckd (Compl)	CATTTATTAGATCTTTAATTTTGTGTTAATCTAGGTCTCGACAACCAGTAGTCATC
TFP-Primer 1	ACGCTAAGCTCGAGCATGGTGAGCAAGGGCGAGGAGACCACAATGGGC
(-4)-BkWd-KpnI(RF1)-TFP	TATCATGGTACCGTTGCGGGCCACGGCGCTCTCGTA
(-3)-BkWd-KpnI(RF1)-TFP	TATCATGGTACCGGAGTTGCGGGCCACGGCGCTCTC
(-2)-BkWd-KpnI(RF1)-TFP	TATCATGGTACCGGTGGAGTTGCGGGCCACGGCG
(-1)-BkWd-KpnI(RF1)-TFP	TATCATGGTACCGTCGGTGGAGTTGCGGGCCACGGC
0-BkWd-KpnI(RF1)-TFP	GCTCATGGTACCGCCGTCGGTGGAGTTGCGGGCCAC
1-BkWd-KpnI(RF1)-TFP	GATATAGGTACCGCTGCCGTCGGTGGAGTTGCGGGCCAC
2-BkWd-KpnI(RF1)-TFP	GATGCAGGTACCTGATCCGCCGTCGGTGGAGTTGCGGGCCAC
3-BkWd-KpnI(RF1)-TFP	GATTAAGGTACCTGTTGATCCGCCGTCGGTGGAGTTGCGGGCCAC
4-BkWd-KpnI(RF1)-TFP	GTATGCGGTACCTGCTGTTGATCCGCCGTCGGTGGAGTTGCGGGCCAC
5-BkWd-KpnI(RF1)-TFP	GTATATGGTACCTGATGCTGTTGATCCGCCGTCGGTGGAGTTGCGGGCCAC
6-BkWd-KpnI(RF1)-TFP	GATCAGGGTACCTGATCCTGCTGTTGATCCGCCGTCGGTGGAGTTGCGGGCCAC

8-BkWd-KpnI(RF1)-TFP	GTAATCGGTACCTGTTCCTGATCCTGCTGTTGATCCGCCGTCGGTGGAGTTGCGGGCCAC
10-BkWd-KpnI(RF1)-TFP	CATATAGGTACCTGCTCCTGTTCCTGATCCTGCTGTTGATCCGCCGTCGGTGGAGTTGCGGGCCAC
14-BkWd-KpnI(RF1)-TFP	TTTAATGGTACCTGCTGTACCTGATGCTCCTGTTCCTGATCCTGCTGTTGATCCGCCGTCGGTGGAGTTGCGGGCCAC
(-3)-FrWd-EagI(RF3)-YFP	CTATAATCGGCCGGCACCGGGGTGGTGCCCATCCTGGTCGAG
(-2)-FrWd-EagI(RF3)-YFP	CTATAATCGGCCGGCTTCACCGGGGTGGTGCCCATCCTGGTC
(-1)-FrWd-EagI(RF3)-YFP	CTATAATCGGCCGGCCTGTTCACCGGGGTGGTGCCCATCCTGGTC
0-FrWd-EagI(RF3)-YFP	GTAGCATCGGCCGGCGAGCTGTTCACCGGGGTGGTGCCCATCCTG
1-FrWd-EagI(RF3)-YFP	CTAATATCGGCCGGCAGTGAGCTGTTCACCGGGGTGGTGCCCATCCTG
2-FrWd-EagI(RF3)-YFP	CTGATCTCGGCCGGCGGTAGTGAGCTGTTCACCGGGGTGGTGCCCATCCTG
3-FrWd-EagI(RF3)-YFP	CTAATCTCGGCCGGCTCAGGTAGTGAGCTGTTCACCGGGGTGGTGCCCATCCTG
4-FrWd-EagI(RF3)-YFP	GATACATCGGCCGGCGCAACGGGTAGTGAGCTGTTCACCGGGGTGGTGCCCATCCTG
6-FrWd-EagI(RF3)-YFP	CTATCATCGGCCGGCGGAAGCGCAACGGGTAGTGAGCTGTTCACCGGGGTGGTGCCCATCCTG
8-FrWd-EagI(RF3)-YFP	CAATAATCGGCCGGCACGGCTGGAAGCGCAACGGGTAGTGAGCTGTTCACCGGGGTGGTGCCCATCCTG
10-FrWd-EagI(RF3)-YFP	CATATATCGGCCGGCACGGGAACGGCTGGAAGCGCAACGGGTAGTGAGCTGTTCACCGGGGTGGTGCCCATCCTG
14-FrWd-EagI(RF3)-YFP	TATTTATCGGCCGGCGCTGGCAGTGGAACGGGAACGGCTGGAAGCGCAACGGGTAGTGAGCTGTTCACCGGGGTGGTGCCCATCCTG
YFP-Primer 4	GTCTGCAAGCTTCTTACTTGTACAGCTCGTCCATGCCGAGAGTGATCCCGGCG
LigCbx7-H3K27-forward	GATAGCGGTACCATGGAGCTGTCA
LigCbx7-H3K27-Backward	GATGATAGCCGGCCGTTCCTGTAG
SalI(RF2)-H3K27-Sense	CATGTAGGGTCGACACTTGCTACAAAGGCAGCACGCAAGAGCGCACCTGCTACAGGAACGGCCGGCTATCATC
EagI(RF3)-H3K27-Antisense	GATGATAGCCGGCCGTTCCTGTAGCAGGTGCGCTCTTGCGTGCTGCCTTTGTAGCAAGTGTCGACCCTACATG
FrWd-KpnI(RF1)-Cbx7	GATAGCGGTACCATGGAGCTGTCAGCCATAGGCGAGCAGGTG
0-BkWd-SalI(RF2)-Cbx7	GGAACCTGTCGACCCCTCCTCCTTCTCCTCGTAGGCCATGACAAGGCG
2-BkWd-SalI(RF2)-Cbx7	AGTCGCTGTCGACCCTGATCCCTCCTCCTTCTCCTCGTAGGCCATGACAAGGCG
4-BkWd-SalI(RF2)-Cbx7	GTGGCGTGTCGACCCTGCTGTTGATCCCTCCTCCTTCTCCTCGTAGGCCATGACAAGGCG
6-BkWd-SalI(RF2)-Cbx7	GCTTCGTGTCGACCCTGATCCTGCTGTTGATCCCTCCTCCTTCTCCTCGTAGGCCATGACAAGGCG
8-BkWd-SalI(RF2)-Cbx7	GCTCGATGTCGACCCTGTTCCTGATCCTGCTGTTGATCCCTCCTCCTTCTCCTCGTAGGCCATGACAAGGCG
10-BkWd-SalI(RF2)-Cbx7	GAGCATTGTCGACCCTGCTCCTGTTCCTGATCCTGCTGTTGATCCCTCCTCCTTCTCCTCGTAGGCCATGACAAGGCG
14-BkWd-SalI(RF2)-Cbx7	GTGTACTGTCGACCCTGCTGTACCTGATGCTCCTGTTCCTGATCCTGCTGTTGATCCCTCCTCCTTCTCCTCGTAGGCCATGACAAGGCG
15-BkWd-SalI(RF2)-Cbx7	AGTTACTGTCGACCCTGTTGCTGTACCTGATGCTCCTGTTCCTGATCCTGCTGTTGATCCCTCCTCCTTCTC
20-BkWd-SalI(RF2)-Cbx7	GTTTTATGTCGACCCTCCTGTTCCTCTTCCTGCTGCTGTACCTGATGCTCCTGTTCCTGATCCTGCTGTTGATCCCTCCTCCTTCTCCTCGTAGGCCATGACAAGGCG
25-BkWd-SalI(RF2)-Cbx7	AGTTACTGTCGACCCAGTACCAGCAGATCCTCCTGTTCCTCTTCCTGCTGCTGTACCTGATGCTCCTGTTCCTGA
30-BkWd-SalI(RF2)-Cbx7	AGTTATTGTCGACCCTGCTGTTCCTGATGCAGTACCAGCAGAAGTTCCTGTTCCTCTTCCTGCTGCTGTACCTGATGCTCCTGTTCCTGA
PCAN-F	TCTCGAATGCGGCCGCTAAACTCTAGATAGGGTGGCGGTTTGAGGTG
PCAN-R	AGAGTTTAGCGGCCGCATTCGAGATCTATGACAACTAAAGGAATTGC
JM-F	CGAATTAGATCTGCCTTGCAAAGCATCACTGC
JM-R	CGAATGTCTAGAGGGAAG CTCTTCATCCAGTG

F927-F	TCAGGCTCACCACCGAGACCTNNSATGAAGTCAACTTTG
F927-R	GGTCTCGGTGGTGAGCCTG
G935-S936-F	CCTTCTATGAAGTCAACTTTGATGATNNSNNSTTCAGCGACAATCTTTATC
G935-S936-R	ATCATCAAAGTTGACTTCATAGAAGG
F937-S938-F	GTCAACTTTGATGATGGCTCCNNSNNSGACAATCTTTATCCTG
F937-S938-R	GGAGCCATCATCAAAGTTGAC
D939-N940-F	CTTTGATGATGGCTCCTTCAGCNNSNNSCTTTATCCTGAGGACATAG
D939-N940-R	GCTGAAGGAGCCATCATCAAAG
L941-Y942-F	GATGGCTCCTTCAGCGACAATNNSNNSCCTGAGGACATAGTGAG
L941-Y942-R	ATTGTCGCTGAAGGAGCCATC
E944-D945-F	CCTTCAGCGACAATCTTTATCCTNNSNNSATAGTGAGCCAGGACTG
E944-D945-F	AGGATAAAGATTGTCGCTGAAGG
T968-F	GAAGTGGTCCAAGTGAGATGGNNSGACGGCCAAGTCTATG
T968-R	CCATCTCACTTGGACCACTTC
C71-F	ATGGAGCTGTCAGCCATAGGCGAGCAGGTGTTTGCGGTGGAG
C72-R	GCACGCGCTTCTTCCGGATGCTCTCCACCGCAAACACCTGCTC
C73-F	CATCCGGAAGAAGCGCGTGCGGAAGGGCAAAGTTGAATATCTG
C74-R	GCCATCCTTTCCACTTCACCAGATATTCAACTTTGCCCTTCC
C75-F	GGTGAAGTGGAAAGGATGGCCCCCCAAGTATAGCACCTGGGAGC
C76-R	GTCCAAGATGTGCTCCTCTGGCTCCCAGGTGCTATACTTGGG
C77-F	CCAGAGGAGCACATCTTGGACCCTCGCCTTGTCATGGCCTAC
C78-R	CTCCTCCTTCTCCTCGTAGGCCATGACAAGGCGAGG
C7Bgl-F	AACTTAGATCTATGGAGCTGTCAGCCATAGGCGAG
C7Xba-R	TTCAATCTAGACTCCTCCTTCTCCTCGTAGGCCATG
V25-F	GTCAGCCATAGGCGAGCAGNNSTTTGCGGTGGAGAGC
V25-R	CTGCTCGCCTATGGCTGAC
A27-F	CCATAGGCGAGCAGGTGTTTNNSGTGGAGAGCATCCG
A27-R	AAACACCTGCTCGCCTATGG
K48-F	GCAAAGTTGAATATCTGGTGAAGTGGNNSGGATGGCCCCCCAA
K48-R	CCACTTCACCAGATATTCAACTTTGC
L64-F	GGAGCCAGAGGAGCACATCNNSGACCCTCGCCTTGTC
L64-R	GATGTGCTCCTCTGGCTCC
A71-F	GGACCCTCGCCTTGTCATGNNSTACGAGGAGAAGG
A71-R	CATGACAAGGCGAGGGTCC
H2B-Xho1-F	AATTAACTCGAGAATGCCAGAGCCAGCGAAGTC
H2B-Bgl2-FP-F	TCACCAAGTACACCAGCGCTAAGAGATCTATGGTGAGCAAGGGCGAG
H2B-Bgl2-FP-R	CTCGCCCTTGCTCACCATAGATCTCTTAGCGCTGGTGTACTTGGTGA
pBAD-R	CAGGCTGAAAATCTTCTCTCATCCGCC
